# A Novel Drug Candidate for Sepsis Targeting Heparanase by Inhibiting Cytokine Storm

**DOI:** 10.1002/advs.202403337

**Published:** 2024-05-29

**Authors:** Danyang Wang, Kaixuan Wang, Qiutong Liu, Mingyang Liu, Guoqiang Zhang, Ke Feng, Kun Wang, Xianwei Ding, Haomiao Zhu, Song Yang, Yonghui Liu, Tiehai Li, Peng Gong, Manli Wang, Peng George Wang, Hongzhen Jin, Wei Zhao, Fan Yu

**Affiliations:** ^1^ State Key Laboratory of Medicinal Chemical Biology College of Pharmacy Key Laboratory of Molecular Drug Research and KLMDASR of Tianjin Nankai University Tongyan Road, Haihe Education Park Tianjin 300350 China; ^2^ School of Health and Life Sciences Qingdao Central Hospital University of Health and Rehabilitation Sciences Qingdao 266113 China; ^3^ Carbohydrate‐Based Drug Research Center Shanghai Institute of Materia Medical Chinese Academy of Sciences Shanghai 201203 China; ^4^ State Key Laboratory of Virology Wuhan Institute of Virology Chinese Academy of Sciences Wuhan 430071 China; ^5^ School of Medicine Southern University of Science and Technology Shenzhen 518000 China

**Keywords:** cytokine storm, glycocalyx, heparanase, sepsis

## Abstract

Sepsis is an infection‐triggered, rapidly progressive systemic inflammatory syndrome with a high mortality rate. Currently, there are no promising therapeutic strategies for managing this disease in the clinic. Heparanase plays a crucial role in the pathology of sepsis, and its inhibition can significantly relieve related symptoms. Here, a novel heparanase inhibitor **CV122** is rationally designed and synthesized, and its therapeutic potential for sepsis with Lipopolysaccharide (LPS) and Cecal Ligation and Puncture (CLP)‐induced sepsis mouse models are evaluated. It is found that **CV122** potently inhibits heparanase activity in vitro, protects cell surface glycocalyx structure, and reduces the expression of adhesion molecules. In vivo, **CV122** significantly reduces the systemic levels of proinflammatory cytokines, prevents organ damage, improves vitality, and efficiently protects mice from sepsis‐induced death. Mechanistically, **CV122** inhibits the activity of heparanase, reduces its expression in the lungs, and protects glycocalyx structure of lung tissue. It is also found that **CV122** provides effective protection from organ damage and death caused by Crimean‐Congo hemorrhagic fever virus (CCHFV) infection. These results suggest that **CV122** is a potential drug candidate for sepsis therapy targeting heparanase by inhibiting cytokine storm.

## Graphical abstract

Sepsis currently has no specific clinical treatment. The main cause of multi‐organ failure and death in sepsis is increased vascular permeability due to endothelial damage. A drug targeting Heparanase through screening is developed. The drug significantly improved survival rates in sepsis mouse models from 0% to 80%, making it a promising candidate for sepsis.

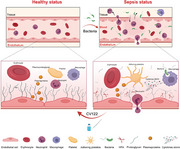

## 1 Introduction

Sepsis is a systemic immune disorder caused by bacterial, fungal, or viral infections.^[^ ^1^ ^]^ Its pathogenesis is often characterized by a “cytokine storm” formed by the rapid release of proinflammatory cytokines and consequent multiorgan dysfunction and failure.^[^ ^2^, ^3^, ^4^ ^]^ Cytokine storm is also a unique immunological feature of COVID‐19, and patients with severe COVID‐19 are highly susceptible to septic shock.^[^ ^5^, ^6^, ^7^ ^]^ In 2020, there were 48.9 million sepsis cases and ≈11 million sepsis‐related deaths worldwide.^[^ ^8^, ^9^ ^]^ The pathogenesis of sepsis is complicated and involves a pathophysiological cascade initiated by infection with pathogenic microorganisms and their toxins in the host.^[^ ^10^ ^]^

Sepsis starts with excessive activation of immune cells and proinflammatory responses, followed by endothelial barrier damage and organ dysfunction.^[^ ^11^ ^]^ Consequently, multiple organs fail and the immune system becomes severely suppressed.^[^ ^12^ ^]^ Given these broad pathophysiological mechanisms contributing to sepsis, several pathways have been considered viable targets for therapeutics: inhibition of pro‐inflammatory responses, alleviation of immunosuppression, prevention of endothelial barrier damage, and alleviation of organ dysfunction.^[^ ^13^ ^]^ Most therapies, however, remain at the research stage, and most drugs with specific single target (such as HA‐1A, eritoran, and tifacogin) have failed in clinical trials with sepsis patients.^[^ ^14^ ^]^ The latest study has found that heparan sulfate octadecasaccharide (18‐mer) could be used as a multi‐target agent to protect against sepsis.^[^ ^15^ ^]^

The vascular endothelial glycocalyx, a carbohydrate‐rich layer lining the vascular endothelium, is one of the earliest sites involved in sepsis. The glycocalyx is composed of glycosaminoglycans (GAGs), proteoglycans (PGs), sialic acid‐binding glycoproteins (GPs), and related plasma glycoproteins.^[^ ^16^ ^]^ As the vascular endothelial barrier, glycocalyx plays an important role in modulating vascular permeability, regulating inflammatory responses, transducing vascular mechanical shear force, and anticoagulation. Sepsis is directly related to the destruction of endothelial glycocalyx as it is one of the first targets affected during the inflammatory process. When inflammation occurs, the body releases a large amount of inflammatory mediators, enzymes, and active substances that impair glycocalyx. These structural disruptions in the endothelium can increase its permeability, allowing plasma proteins and body fluids to cross the blood vessel wall, thereby promoting edema formation and multiorgan dysfunction.^[^ ^17^ ^]^ Meanwhile, the degraded glycocalyx components, including intercellular cell adhesion molecule‐1 (ICAM‐1), vascular cell adhesion molecule (VCAM‐1), HS, HA, and syndecan‐1, are released into the plasma, leading to uncontrolled activation of the immune system and severe cytokine storm.^[^ ^18^ ^]^ Therefore, the degree of glycocalyx degradation is an indicator of the severity of sepsis, making it an attractive biomarker and therapeutic target in sepsis‐related multiple organ failure.^[^ ^19^, ^20^ ^]^

Glycocalyx degradation is mainly mediated by heparanase (HPA), the endoglycosidase that can hydrolyze heparan sulfate (HS) in mammals. HPA is highly expressed only in the placenta and certain blood cells (including platelets, neutrophils, mast cells, and lymphocytes) under normal physiological conditions.^[^ ^21^ ^]^ However, in particular pathological circumstances such as sepsis, HPA is activated by reactive oxygen species (ROS) and pro‐inflammatory cytokines such as TNF‐α, IL‐1β, and IL‐6, and also overexpressed in endothelial and epithelial cells in inflammatory diseases.^[^ ^11^ ^]^ In acute lung injury of sepsis, pulmonary capillary endothelial cells quickly activate endogenous HPA, and the activated HPA severs HS on the glycocalyx of lung endothelial cells, resulting in endothelial cell glycocalyx destruction.^[^ ^22^ ^]^ Therefore, HPA inhibitors are attractive candidates for the prevention and treatment of sepsis.

**Scheme 1:**
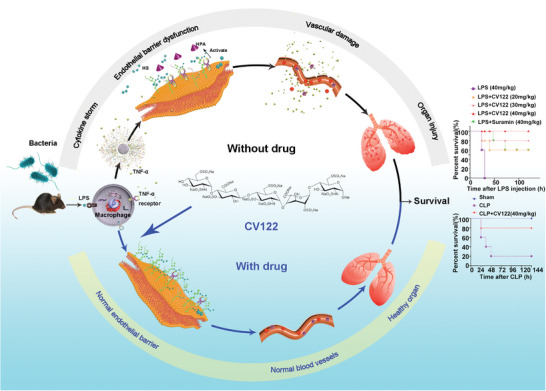
Schematic of the study. First, a mouse model of sepsis was established with the Gram‐negative bacterial endotoxin LPS, which activates the mouse's innate immune system, dysregulating immune responses such as the macrophage and causing abundant cytokine release. This “storm” activates endothelial cells to release HPA, which degrades HS in the vascular endothelial glycocalyx, thereby increasing vascular permeability. Tissue and plasma proteins extravasate into surrounding tissues, lungs, and other organs are damaged, and eventually, the mice die. When septic mice are infected and administered **CV122**, **CV122** acts as an HPA inhibitor, reducing the degradation of HS in the vascular endothelial glycocalyx, thereby maintaining the vascular barrier. **CV122** ameliorates multi‐organ damage and ultimately improves the survival rate of septic mice. Similarly, **CV122** was shown to improve mouse survival in the CLP model.

Several recent studies proved that heparin oligosaccharide derivatives could be used as HPA inhibitors in several diseases such as tumors, but whether or what kind of heparin oligosaccharide derivatives could be potential drug candidates for sepsis remains to be investigated. In this study, based on our previous work about the heparin pentasaccharide fondaparinux^[^ ^23^, ^24^, ^25^ ^]^ and the associated work on synthesis of heparin oligosaccharides,^[^ ^26^, ^27^ ^]^ we designed and synthesized small molecule HPA inhibitors through structure modification of fondaparinux and further screened **CV122** as one of the most effective inhibitors of HPA in vitro, in cellulo and in vivo, which could protect the vascular endothelial cell glycocalyx and relieve sepsis. **CV122** was proved to efficiently inhibit the activity and expression of HPA, and protect the surface glycocalyx of macrophages and endothelial cells. **CV122** also showed an excellent protective effect in LPS and CLP‐induced septic mice by increasing the survival rate from 0% to 80%. The underlying mechanism of **CV122** against sepsis was further investigated by the evaluation of its effects on inflammation, organ damage, sepsis‐related immunosuppression, HPA levels, and cell surface glycocalyx in lung tissues. Finally, we used the CCHFV infected mouse model, which also leads to severe blood vessel damage similar to sepsis,^[^ ^28^ ^]^ to verify whether **CV122** could protect the blood vessel and organ damages caused by the viral infection. Overall, **CV122** is a promising candidate for blood vessel protection and sepsis against various infections by inhibiting cytokine storm and now requires further preclinical and clinical development (**Scheme** **1**).

## 2 Results

### 2.1 Screening of Candidates

**Figure 1:**
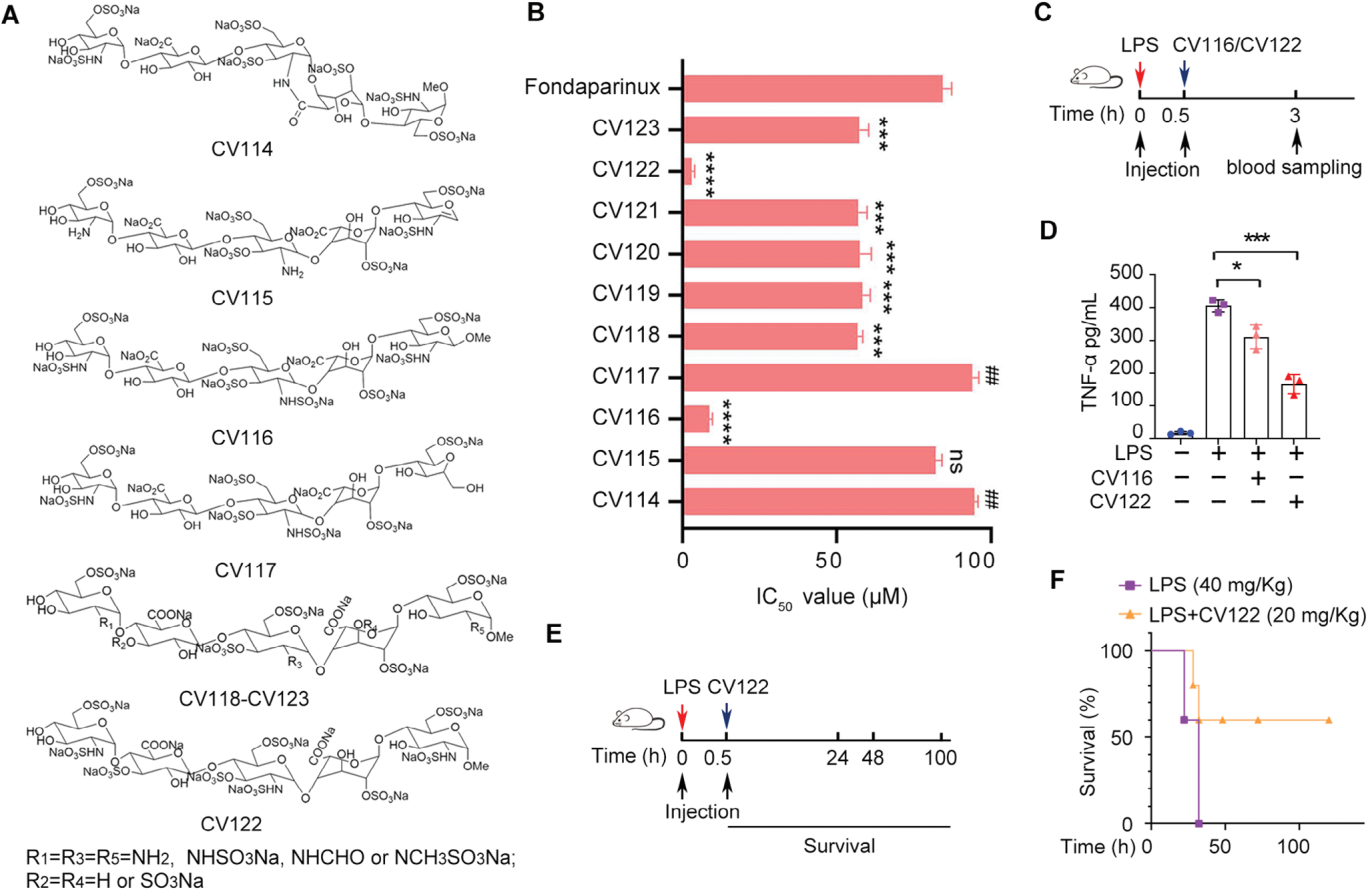
Structure of the synthetic oligosaccharides and screening of candidates. A) Structure of **CV114**‐**CV123**. B) Inhibition of HPA by **CV114**‐**CV123** and Fondaparinux in vitro. C) A schematic of the sepsis model and administration methods with **CV116/CV122** and blood sampling. D) TNF‐α in the serum of mice. E) A schematic of the sepsis model and administration methods with **CV122**. F) The effect of the drug candidate **CV122** (20 mg Kg^−1^) on the survival rate of septic mice. *p < 0.05, **p < 0.01, ***p < 0.001, ****p < 0.0001, ##*p* < 0.01, ns, not significant. Error bars indicate SD.

We assumed that HPA could be a promising target to treat sepsis by inhibiting the disruption of glycocalyx. Thus, we designed and synthesized a series of structural analogs of its substrate. Ten pentasaccharide candidates **CV114**‐**CV123** were synthesized through structure modification of fondaparinux, an FDA‐approved drug (**Figure** **1**A). Then the activities of HPA inhibition were tested and **CV122** showed the lowest IC_50_ values compared with fondaparinux (****p < 0.0001) (Figure 1B). Detailed methods for the synthesis and characterization of **CV122** are shown in Figures S1, S1‐1, and S2 (Supporting Information). We then examined the effect of **CV122** with an LPS‐induced septic mouse model and **CV122** showed excellent anti‐inflammatory activity by significantly decreasing TNF‐α levels in serum (Figure 1C,D). Furthermore, **CV122** also efficiently improved the survival rate of the LPS‐induced mouse model from 0% to 60% (Figure 1E,F).

### 2.2 CV122 Improves the Survival of LPS‐induced Septic Mice

**Figure 2:**
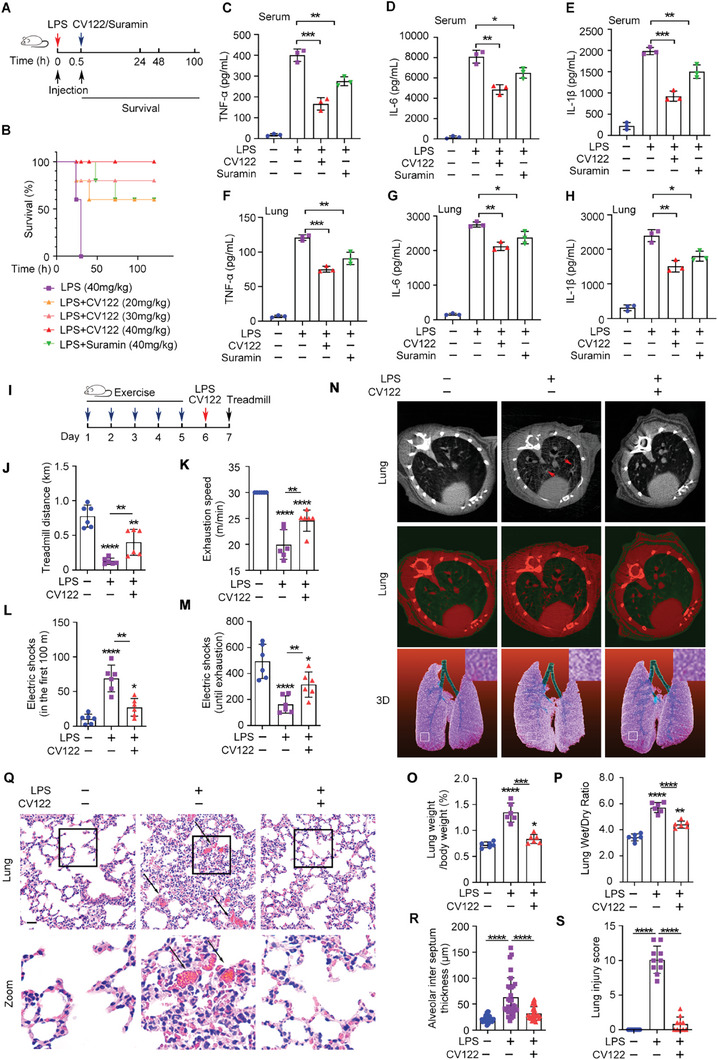
Mouse survival, inflammatory factors and organ damage in LPS‐induced septic mice with or without **CV122** treatment. A) Schematic of the mouse sepsis model and administration methods. B) The effect of **CV122** on the survival rate of septic mice, (*n =* 5). C–H) TNF‐α, IL‐6, and IL‐1β in the serum and lungs of mice. I) Schematic of mouse vitality experiments. J,K) Mouse running distance and exhaustion speed. L,M) Mouse running distance by electric shocks in the first 100 m and until exhaustion. N–P) CT imaging of mouse lungs and lung weight/body weight and lung wet–dry ratios. Q–S) H&E‐stained lung tissue sections and histopathological evaluation of alveolar inter‐septal thickness and lung injury score. Scale bars, 100 µm. *p < 0.05, **p < 0.01, ***p < 0.001, ****p < 0.0001; ns, not significant. Error bars indicate SD.

To further estimate the effect of **CV122**, we treated the LPS‐induced septic mice with different doses (**Figure** **2**A). While 20 mg kg^−1^ of **CV122** efficiently increased the survival rate up to 60%, 40 mg kg^−1^ of **CV122** could completely protect the mice from death over an 80 h or longer observation period. We used Suramin, a previously reported HPA inhibitor with a mainly noncompetitive inhibitory effect,^[^ ^29^, ^30^ ^]^ as a positive comparison. The same dose of Suramin (40 mg kg^−1^) achieved a survival rate of 60% compared with 100% of **CV122**, suggesting **CV122** was a much more effective drug candidate for sepsis (Figure 2B). These results indicate that **CV122** could effectively improve the survival of septic mice in a dose‐dependent manner and to a higher degree than Suramin.

In the meantime, **CV122** inhibited cytokine release. The blood and lungs were collected 3 h after LPS injection, and the levels of pro‐inflammatory cytokines TNF‐α, IL‐6, and IL‐1β were quantified (Figure 2C–H). Serum and lung TNF‐α, IL‐6, and IL‐1β were very low in untreated mice and rapidly and significantly increased after LPS injection, consistent with cytokine storm. Compared with the LPS‐stimulated group, **CV122** significantly suppressed TNF‐α, IL‐6 and IL‐1β levels. Suramin could also suppress the cytokine release but to a lesser extent. TNF‐α, IL‐6, and IL‐1β levels were also measured in the tissue extracts of brains, hearts, livers, kidneys, and spleens (Figure S3, Supporting Information), and **CV122** significantly inhibited the secretion of proinflammatory cytokines in these organs to a greater extent than Suramin.

To further assess the protective effect of **CV122** in LPS‐induced septic mice, we assessed the vitality of the mice with or without **CV122** treatment by treadmill (Figure 2I). **CV122** significantly increased the running distance and extended the time to exhaustion of LPS‐treated mice (Figure 2J,K). Likewise, **CV122** reduced the electric shocks needed in the first 100 m and obviously increased the total electric shocks until exhaustion, indicating the improvement of vitality after **CV122** treatment (Figure 2L,M). Taken together, these data suggest that **CV122** effectively improved the healthy status of LPS‐induced septic mice.

### 2.3 CV122 Prevents Organ Damages in LPS‐induced Septic Mice

Sepsis is often accompanied by multi‐organ failure, so we next examined the protective effect of **CV122** in LPS‐induced mouse organs. As the lung is usually the most vulnerable organ during sepsis, we first checked the lung damage by micro‐CT imaging. While the lung developed severe inflammation with LPS injection, **CV122** could almost completely inhibit this progress (Figure 2N). After administration of **CV122**, the white stripes indicating lung texture induced by LPS were partially decreased, suggesting that **CV122** protected vascular endothelial cells from damage and decreased exudate.

**Figure 3:**
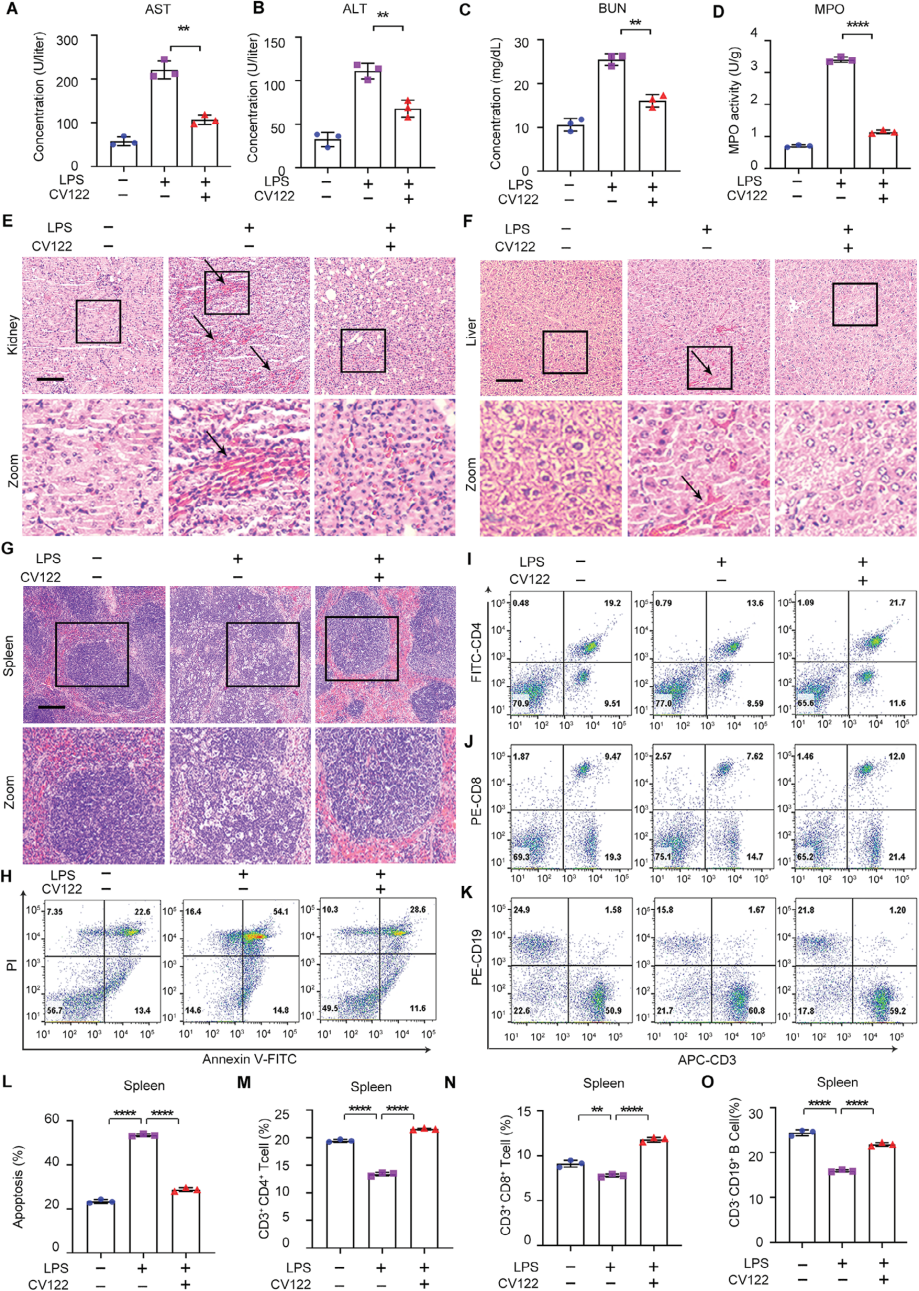
Organ damage and immunosuppression in LPS‐induced septic mice. A–D) ALT, AST, BUN in serum of mice, MPO activity in lung tissue. E–G) H&E for histopathological evaluation of Liver, Kidney, and Spleen. H,L) Splenocyte apoptosis was detected by flow cytometry using FITC‐Annexin V and PI staining, I,M) Flow cytometric detection of CD3^+^CD4^+^T cells in spleens. J,N) Flow cytometric detection of CD3^+^CD8^+^T cells in spleens. K,O) Flow cytometric detection of CD3^−^CD19^+^B cells in spleens. Scale bars, 100 µm. *p < 0.05, **p < 0.01, ***p < 0.001, ****p < 0.0001; ns, not significant. Error bars indicate SD.

This result was further proved by the lung weight/body weight ratio and the wet–dry weight ratio of the lung. The ratio is a direct indicator of reactive pulmonary edema and a sensitive response to the severity of lung injury. After the onset of acute lung injury, massive fluid seeps into the alveoli and the pulmonary interstitium, resulting in increased lung weight, while the dry weight of the lung is unaffected. According to the statistical results (Figure 2O,P), both ratios were significantly increased in septic mice compared with the control mice (****p < 0.0001), however, with **CV122** treatment, the weight/body weight ratio of the lung was significantly decreased (***p < 0.001) and the wet–dry‐weight ratio of the lung was extremely significantly decreased (****p < 0.0001), suggesting less inflammation and injury of the lung upon **CV122** protection. These results indicate that **CV122** could well improve the acute lung injury caused by sepsis. Pathological damage in mouse organs was next assessed in H&E‐stained tissue sections. In mice administered LPS, the lungs showed severe congestion, inflammatory cell infiltration, alveolar wall thickening, and interstitial edema. These features were significantly improved by **CV122** administration (Figure 2Q). The histopathological evaluation of alveolar inter‐septal thickness and organ injury score further proved this result (Figure 2R,S). Similarly, kidney damage caused by LPS was evidenced by the expansion of renal tubules, degeneration of vacuoles, and missing brush margins, which again were resolved by **CV122** (**Figure** **3**E). LPS induced obvious liver damage including congestion, swelling, and sinusoidal leukocytosis, and these pathological changes were significantly improved by **CV122** (Figure 3F). In the model group, the pathological characteristics of the spleen were blurred cortical and medulla borders. After treatment with **CV122**, no pathological changes were observed in spleen tissue (Figure 3G). These organ tissue pathological results of mice provided important support for the structural and functional recovery of the organs following treatment with **CV122**.

Mirroring the histopathological findings, indicators of abnormal liver (Aspartate aminotransferase, AST and Alanine aminotransferase, ALT), kidney (Urea, BUN), and lung (Myeloperoxidase, MPO) function returned to normal upon **CV122** treatment, which was more effective than Suramin (Figure 3A–D).

### 2.4 CV122 Reduces the Infiltration of Macrophages into the Organs

Macrophages circulating in the blood or in the tissue are the first barrier against external infection. Due to their diversity and plasticity, macrophages undergo heterogeneous activation and polarization. Upon bacterial and viral infection, macrophages undergo M1 differentiation and produce large amounts of inflammatory cytokines, such as IL‐1, IL‐6, TNF‐α, NO, and chemokines, leading to tissue damage, and in general, sepsis is associated with increased expression of M1 markers in mouse monocytes.^[^ ^31^, ^32^ ^]^ To evaluate the therapeutic efficacy of **CV122** on sepsis, M1 macrophages were monitored by flow cytometry. The results are shown in Figure S6, Supporting Information. LPS injection significantly increased M1 macrophages (F4/80^+^CD86^+^, * *p* < 0.001) compared with control mice in the lung, Whereas the percentage of M1 macrophages was significantly reduced by **CV122** (**p < 0.01), Similarly, a significant increase in M1 macrophages was also detected in the kidneys and spleen of septic mice, and **CV122** significantly inhibited the percentage of M1 macrophages.

### 2.5 CV122 Reverses Immunosuppression in LPS‐induced Septic Mice

In the final stage of severe sepsis, massive cell apoptosis occurs, especially in the spleen, and mice showed immunosuppression. To test whether **CV122** could improve immunosuppression, we used flow cytometry to assess immune organ apoptosis and T/B cell populations in mice with LPS‐induced sepsis. Splenocyte apoptosis was detected and quantified by flow cytometry with FITC‐Annexin V and PI (Figure 3H,L). Severe sepsis significantly increased splenic apoptosis from low baseline levels and **CV122** significantly reduced apoptosis in LPS‐treated mice to near baseline control levels. CD3^+^CD4^+^ T cells and CD3^+^CD8^+^ T cells were also quantified by flow cytometry (Figure 3I,M,J,N), and their number significantly decreased in severe septic mice and was recovered by **CV122** to close to control levels. Similar results were found in CD3^+^CD4^+^ and CD3^+^CD8^+^ T cell populations isolated from the inguinal lymph nodes (Figure S5D,G,F,H, Supporting Information). Similarly, CD3^−^CD19^+^ B cells in the spleen (Figure 3K,O) and CD3^−^CD19^+^ B cells in inguinal lymph nodes of severe septic mice (Figure S5E,I, Supporting Information) were significantly reduced in the LPS group and recovered upon treatment with **CV122**. These results suggest that **CV122** could reverse the immunosuppression by inhibiting the apoptosis of immune cells in the later stage of severe sepsis.

### 2.6 CV122 Inhibits the Activity and Overexpression of HPA In Vitro

**Figure 4:**
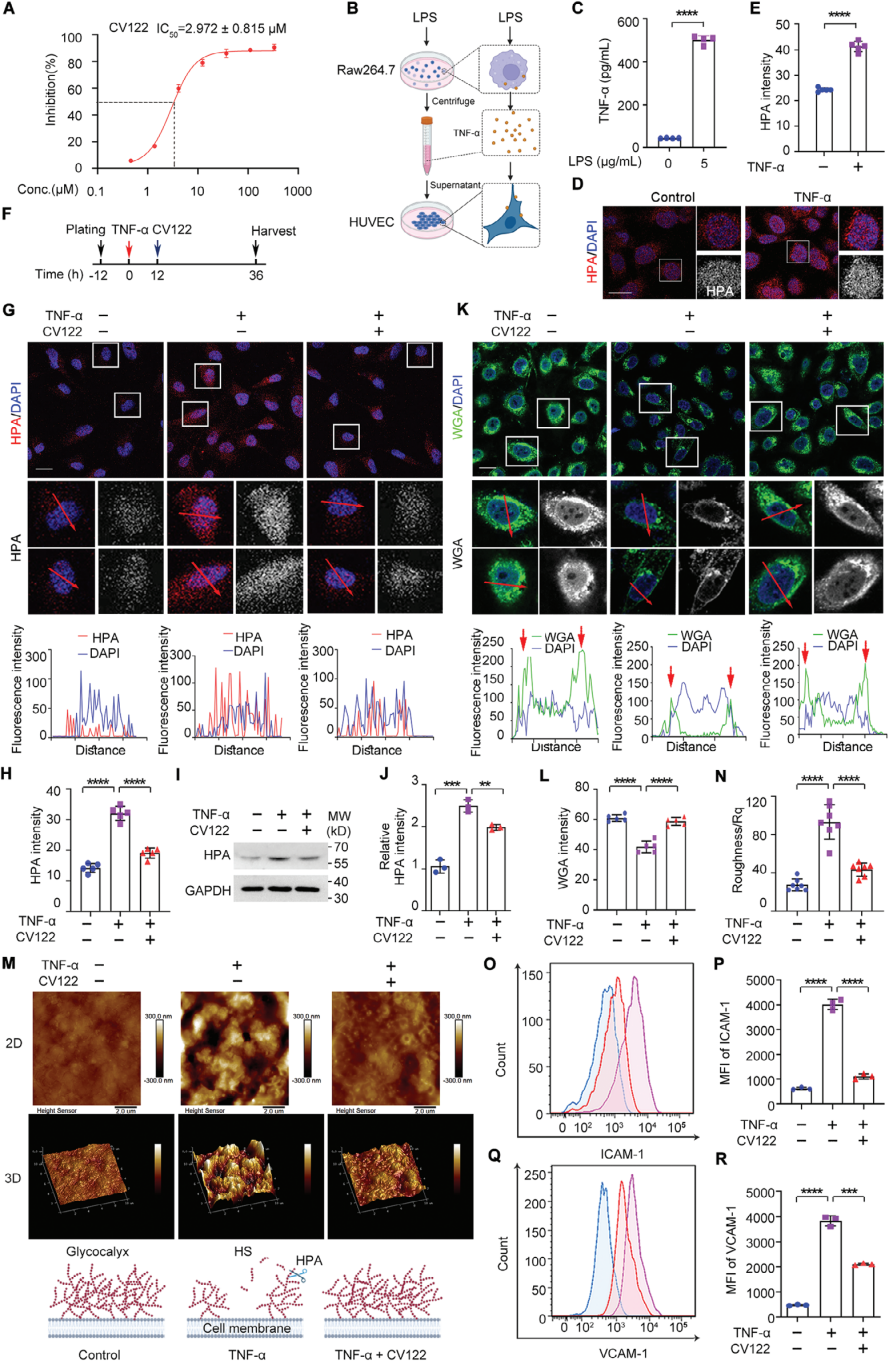
The mechanism of action of **CV122** in HUVECs. A) Inhibition of HPA by **CV122** in vitro. B–E) TNF‐α produced by LPS‐stimulated macrophages increased HPA expression in HUVECs. F) Schematic of the cellular experiments. G,H) HPA levels were detected by immunofluorescence after TNF‐α stimulation of HUVECs. I,J) Western blotting was used to detect HPA protein levels in HUVECs, with GAPDH used as a housekeeping control. K,L) Laser confocal microscopy was used to detect changes in WGA‐FITC fluorescence intensity in each group and after **CV122** treatment of HUVECs. M,N) Atomic force microscopy was used to examine the effect of **CV122** on the cell surface glycocalyx structure of HUVECs. 2D and 3D structures on the cell surface were observed. O–R) Expression of adhesion molecules ICAM‐1 and VCAM‐1 were detected by flow cytometry after TNF‐α stimulation of HUVECs. Scale bars, 20 µm. **p < 0.01, ***p < 0.001, ****p < 0.0001; ns, not significant. Error bars indicate SD.

We next sought to understand the underlying mechanism of how **CV122** protects mice against sepsis. We used homogeneous time‐resolved fluorescence (HTRF) to detect the inhibition effect of **CV122** on HPA. As shown in **Figure** **4**A, the IC_50_ value of **CV122** was 2.97 ± 0.82 µM, suggesting that **CV122** strongly inhibited HPA activity in vitro. To further investigate the mechanism of **CV122**, we established in cellulo models of LPS‐induced cytokine release, in which TNF‐α produced by LPS‐stimulated macrophages increased HPA levels in human umbilical vein endothelial cells (HUVECs) (Figure 4B–E). Immunofluorescence and western blotting were used to find out the effect of **CV122** on HPA protein expression in endothelial cells. As shown in Figure 4G–J, **CV122** significantly inhibited HPA expression in HUVECs. HPA levels were increased by TNF‐α in HUVECs, and **CV122** treatment significantly reduced HPA overexpression. This result was further confirmed by western blotting. Similarly, **CV122** significantly inhibited HPA levels following LPS stimulation of macrophages, as measured by both immunofluorescence and western blotting (Figure S4B–E, Supporting Information). These results reveal the molecular mechanism that **CV122** could inhibit both the activity and overexpression of HPA induced by cytokine and therefore prevent downstream consequences.

### 2.7 CV122 Protects the Cell Surface Glycocalyx from Damage In Vitro

Activation and overexpression of HPA lead to glycocalyx damage during sepsis. To detect whether the cell surface glycocalyx could be protected by **CV122**, we stimulated HUVECs with TNF‐α and then examined the glycocalyx with wheat germ agglutinin (WGA)‐FITC. WGA can specifically bind cells to GlcNAc in the glycocalyx on the cell surface, and its content is directly proportional to the integrity of the glycocalyx.^[^ ^33^, ^34^ ^]^ Uniform green fluorescence on the cell surface of controls indicated an intact glycocalyx (Figure 4K,L), while the reduction in cell membrane fluorescence in cells stimulated with TNF‐α indicated glycocalyx degradation. Treatment with **CV122** rescued glycocalyx integrity as visualized with WGA‐FITC. Similarly, **CV122** significantly protected the glycocalyx of macrophages upon LPS stimulation (Figure S4F,G, Supporting Information).

To further visualize the changes in glycocalyx structure, we used atomic force microscopy to examine the surface roughness. Normal cells showed a relatively smooth surface in both 2D and 3D views while after TNF‐α stimulation, the glycocalyx structure was destroyed and the cell surface appeared partially collapsed. **CV122** administration reduced glycocalyx damage and resulted in a smoother surface, and roughness *R*_q_ values for each group were consistent with the degree of glycocalyx damage (Figure 4M,N). Therefore, **CV122** effectively inhibits glycocalyx destruction caused by TNF‐α stimulation. Similarly, we found that **CV122** also protected against disruption of the glycocalyx structure following LPS stimulation of macrophages (Figure S4H,I, Supporting Information).

Disruption of glycocalyx leads to the exposure of surface adhesion molecules, which allows the immune cells in the blood to attach to the vessels and then enter the tissues, resulting in inflammation. Flow cytometry was next used to detect ICAM‐1 and VCAM‐1 adhesion molecules on the cell surface of HUVECs (Figure 4O–R) and RAW264.7 macrophages (Figure S4J–M, Supporting Information). TNF‐α‐stimulated HUVECs and LPS‐stimulated RAW264.7 macrophages increased ICAM‐1 and VCAM‐1 expression, which was reversed after **CV122** treatment. Taken together, these results indicate that **CV122** could efficiently protect the cell surface glycocalyx and reduce the exposure of surface adhesion molecules, therefore preventing subsequent inflammation.

### 2.8 CV122 Inhibits HPA Overexpression and Protects the Cell Surface Glycocalyx in Lung Tissue in LPS‐induced Septic Mice

**Figure 5:**
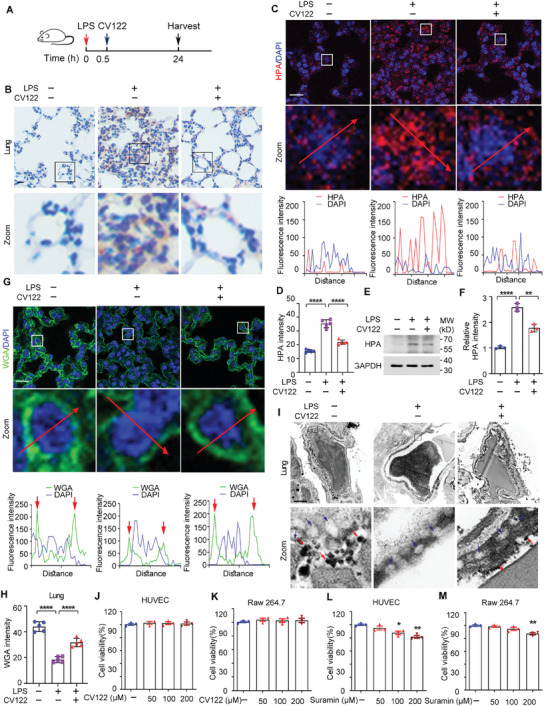
The mechanism of action of **CV122** in lungs of severe LPS‐induced septic mouse. A) Schematic of the mouse experiments. B) Mouse lung tissue sections were stained with anti‐HPA antibodies to detect HPA expression. Scale bars, 20 µm. C,D) Mouse lung tissue sections were stained with HPA and DAPI to detect HPA levels by laser confocal microscopy. Scale bars, 20 µm. E,F) Western blotting was used to detect HPA protein levels in the lungs, and GAPDH was used as a housekeeping control. G,H) Mouse lung tissue sections were stained with WGA and DAPI to detect the cell surface glycocalyx by laser confocal microscopy. Scale bars, 20 µm. I) Detection of glycocalyx on the surface of mouse pulmonary vascular endothelium by transmission electron microscopy. Scale bars, 0.25 µm. J–M) CCK‐8 kit detects the effects of **CV122** and Suramin at different concentrations (50, 100, and 200 µM) on the survival rate of RAW264.7 and HUVEC. *p < 0.05, **p < 0.01, ***p < 0.001, ****p < 0.0001; ns, not significant. Error bars indicate SD.

To further investigate the mechanism of action of **CV122**, LPS‐induced sepsis was again established in mice, as shown in **Figure** **5**A. Lungs were harvested 24 h after LPS injection and HPA expression was assessed by immunohistochemistry. **CV122** significantly reduced the HPA overexpression induced by LPS in the lungs (Figure 5B). Similar results were found in liver and kidney tissue sections (Figure S5B,C, Supporting Information), and this result was confirmed by immunofluorescence and immunoblotting (Figure 5C–F).

To further investigate the protective effect of **CV122** on the cell surface glycocalyx of tissues in septic mice, we visualized the cell surface glycocalyx in the lung tissues by immunofluorescence with WGA‐FITC (Figure 5G,H). Uniform green fluorescence on the cell surface of the control group showed an intact glycocalyx, whereas reduced membrane fluorescence on the cells in the lung tissue of septic mice indicated glycocalyx degradation. The integrity of the glycocalyx was rescued after **CV122** treatment.

In addition, we used the lanthanum nitrate tracing method with transmission electron microscopy to investigate the effect of **CV122** on mouse lung vascular endothelial cell surface glycocalyx protection. The surface of control endothelial cells (blue arrow) stained with lanthanum nitrate (red arrow), and septic mice showed almost no endothelial cell staining but **CV122** significantly restored lanthanum nitrate staining (Figure 5I). These results suggest that **CV122** clearly protects the surface glycocalyx of mouse pulmonary vascular endothelial cells in vivo.

### 2.9 CV122 Improves the Survival of and Prevents Organs From Damage in CLP‐induced Septic Mice

**Figure 6:**
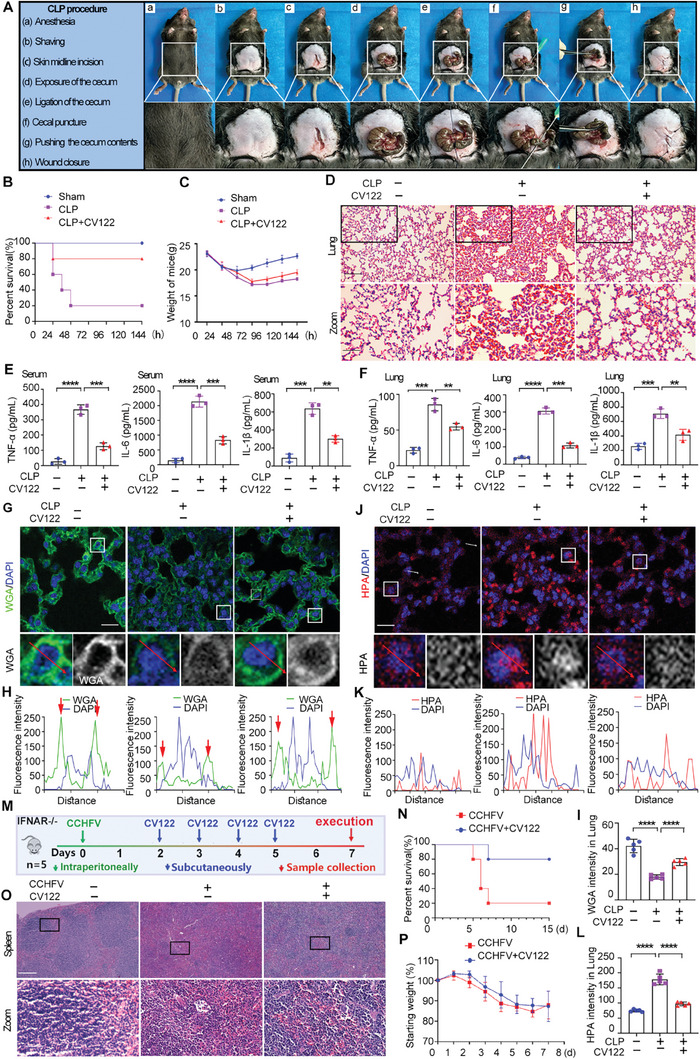
The therapeutic effect of **CV122** in CLP‐induced septic mice and CCHFV‐infected mice. A) CLP procedure. B) The effect of **CV122** on the survival rate of CLP‐induced septic mice, (*n =* 5). C) The effect of **CV122** on the body weight of CLP‐induced septic mice. D) H&E‐stained lung tissue sections. Scale bars, 100 µm. E,F) TNF‐α, IL‐6, and IL‐1β in the serum and lungs of mice. G–I) Mouse lung tissue sections were stained with WGA and DAPI to detect the cell surface glycocalyx by laser confocal microscopy. Scale bars, 20 µm. J–L) Mouse lung tissue sections were stained with HPA and DAPI to detect HPA levels by laser confocal microscopy. Scale bars, 20 µm. M) Flow chart of drug treatment with **CV122** in CCHFV‐infected mice. N) The effect of **CV122** on the survival rate of CCHFV‐infected mice, (*n =* 5). O) H&E‐stained spleen tissue sections. Scale bars, 500 µm. P) The effect of **CV122** on the body weight of CCHFV‐infected mice. *p < 0.05, **p < 0.01, ***p < 0.001, ****p < 0.0001; ns, not significant. Error bars indicate SD.

To further examine the protective effect of **CV122** in sepsis, we built a CLP‐induced septic mouse model, which developed symptoms 30 min after the CLP procedure (**Figure** **6**A). **CV122** also provided efficient protection against CLP‐induced sepsis by increasing the survival rate from 20% to 80% over a 144 h or longer observation period (Figure 6B). The body weight of the mice gradually decreased after surgery, reaching a minimum level at 72 h, and then slowly recovered (Figure 6C), demonstrating that **CV122** gradually normalized the septic mice after the acute infection. We stained lung tissue sections with HE to assess pathological damage in mouse organs. The CLP group showed severe lung congestion, inflammatory cell infiltration, alveolar wall thickening, and interstitial edema, which were significantly rescued by administration of **CV122** (Figure 6D). **CV122** also reduced the level of TNF‐α, IL‐6, and IL‐1β in the serum and lungs of mice, indicating less inflammatory symptoms (Figure 6E,F).

In accordance with LPS‐induced sepsis, **CV122** significantly reduced the HPA levels in the lungs and protected cell surface glycocalyx (Figure 6J–I). These results further confirmed the protective effect of **CV122** against sepsis.

### 2.10 CV122 Improves the Survival of and Prevents Organs from Damage in CCHFV‐Infected Mice

To test whether **CV122** could also be used to treat other diseases accompanied by severe blood vessel damage and organ failure, we examined the effect of **CV122** in CCHFV‐infected mice (Figure 6M). Surprisingly, **CV122** also showed excellent protection against CCHFV infection by increasing the survival rate from 20% to 80% over 8 day or longer observation period. Although the body weights of the mice were decreased after infection, most mice survived upon **CV122** treatment with slightly higher body weights (Figure 6P). We stained spleen tissue sections with H&E to assess pathological damage in mouse organs. The CCHFV group showed severe spleen damage including spleen hemorrhage and edema, disappeared white and medulla boundaries, disintegrated splenosome structure, lymphocyte necrosis, and macrophages and monocyte lymphocyte hyperplasia in medulla part. Upon **CV122** treatment, the spleen congestion and edema were slightly reduced, the boundary between white and medulla was blurred, the splenosomes became smaller or even disappeared, lymphocytes were necrotic and the number of lymphocytes was significantly reduced; the macrophages and monocyte lymphocytes in the whole disease was slightly lighter than that of the CCHFV group (Figure 6O).

### 2.11 Safety Evaluation of CV122

**CV122** was proved to be a promising drug candidate for sepsis. In order to assess its possibility for application in the clinic, cytotoxicity testing was performed in vitro (Figure 5J–M). **CV122** had no significant inhibitory effect on the survival of HUVECs and RAW264.7 macrophages at doses between 50 and 200 µM, indicating that **CV122** was non‐toxic at this dose. As a comparison, 100 and 200 µM Suramin showed a significant inhibitory effect on the survival of HUVECs (*p < 0.05 and **p < 0.01), and 200 µM Suramin showed significant inhibitory effect on RAW264.7 macrophages (**p < 0.01), suggesting that Suramin is relatively cytotoxic while **CV122** is much safer and has great potential as a drug candidate.

## 3 Discussion

As a widely distributed disease with extremely high mortality worldwide, sepsis has a scarce clinical treatment. Since a variety of factors contribute to the pathology of sepsis, identifying the most important targets presents a strategic challenge to discover effective therapeutic interventions. Despite the multifaceted advances in inhibiting the generation or isolation of toxic factors that contribute to sepsis pathology, no recent treatments have significantly improved survival. Therefore, it remains a great urge and challenge to develop treatments and drugs for sepsis.

We found that HPA plays a critical role in the pathogenesis of sepsis.^[^ ^11^ ^]^ The massive release of inflammatory factors caused by sepsis activates HPA in endothelial cells and epithelial cells. Activated HPA increases HS degradation and destruction of vascular endothelial glycocalyx, leading to capillary damage, leakage, and ultimately organ damage. The disrupted glycocalyx further stimulates the expression and activity of HPA, forming a vicious cycle of “HPA activation‐glycocalyx disruption‐HPA activation” and ultimately resulting in an uncontrolled cytokine storm. Therefore, HPA occupies the core position and HPA inhibitors may be effective treatments for sepsis. Based on the strategy of substrate analogs inhibition of the enzyme, several oligosaccharide inhibitors targeting HPA have been designed in the previous research, including SST001,^[^ ^35^ ^]^ M402,^[^ ^36^ ^]^ and PI88.^[^ ^37^ ^]^ These inhibitor drugs have entered clinical research for the treatment of tumors in which HPA is overexpressed, which provides great confidence in our research to develop oligosaccharide inhibitors for sepsis.

Here we rationally designed and synthesized ten oligosaccharide HPA inhibitors based on the structure‐activity relationship between the crystal structure of the HPA protein and its substrate. We found that **CV122** had the lowest IC_50_ values of 2.97 ± 0.82 µm, which might be due to enhanced binding by glucuronic sulfonate at position 3 of **CV122** and a higher degree of sulfation increased affinity for the enzyme, indicating **CV122** has strong HPA inhibitory activity.

**CV122** has been proved in this study to efficiently alleviate sepsis in three models with endotoxin, bacterial, and viral infections. In LPS‐induced sepsis, **CV122** significantly suppressed the release of early cytokines, protected the glycocalyx structure of vascular endothelial cells, protected against organ damage, improved vitality, and raised the survival rate from 0% to 100%. In addition to the LPS model, CLP model in rodents has become the most widely used model for experimental sepsis and is currently considered as the gold standard in sepsis research.^[^ ^38^, ^39^, ^40^ ^]^ **CV122** also showed a satisfying protective effect in CLP model by improving survival from 20% to 80%. Besides, we assumed that **CV122** might be also effective in other diseases with similar pathogenesis. A common pathogenic feature of CCHFV infection is the destruction of blood vessels similar to sepsis.^[^ ^41^ ^]^ We applied **CV122** to this disease and found that **CV122** successfully improved its survival from 20% to 80%. However, it has not been discovered whether CCHFV infection is also related to HPA activation or glycocalyx disruption, which could be further explored next and we hypothesized that **CV122** is responsible for inhibiting the disruption of the glycocalyx on the surface of endothelial cells by inhibiting HPA expression. Other infection‐caused diseases with the pathological features of blood vessel disruption and multi‐organ bleeding and dysfunction might also be among the application areas of **CV122**. Furthermore, many diseases have been proven to have increased HPA activity and/or expression, such as cancer and rheumatoid arthritis, hepatitis C infection, chronic pancreatitis, and ulcerative colitis.^[^ ^42^, ^43^ ^]^ It is reasonable to believe that **CV122** could be applicable in these diseases, which makes it a promising drug candidate with great potential.

Several challenges remain to be overcome in this research. First, the massive synthesis of ten oligosaccharide inhibitors of HPA is a time‐consuming task and the synthesis route needs to be further optimized. Secondly, the drug candidate has only been tested with mouse models and needs further verification of drug trials in monkeys or in patients in clinical practice.

## 4 Experimental Section

### Cell Culture

HUVECs and mouse RAW264.7 macrophages were obtained from the American Type Culture Collection (ATCC). Cells were cultured in Dulbecco's modified Eagle's medium (DMEM) supplemented with 10% (v/v) heat‐inactivated FBS in a humidified incubator at 37 °C and 5% CO_2_/95% air (v/v). All media were supplemented with 10% FBS and 100 units mL^−1^ penicillin/streptomycin.

### Antibodies and Reagents

Rb a Heparanase 1 (Bioss, bs‐1541R); GAPDH Monoclonal Antibody (Immuno Way, YM3029); Goat Anti‐Rabbit IgG H&L (Alexa Fluor 594) (abcam, ab150080); Goat Anti‐Rabbit IgG (Elabscience, E‐AB‐1034); Goat Anti‐Mouse IgG (Elabscience, E‐AB‐1035); APC anti mouse CD3 (Biolegend, 100235); FITC anti mouse CD4 (Biolegend, 100509); PE anti mouse CD8a (Biolegend, 100707); PE anti mouse CD19 (Biolegend, 115507); FITC Anti‐mouse CD54 antibody (Elabscience, E‐AB‐F1018C); FITC Anti‐Human CD54 antibody (Elabscience, E‐AB‐F1043C); FITC Anti‐mouse CD106 antibody (Elabscience, E‐AB‐F1091M); FITC‐WGA Lectin (GeneTex, GTX01502); Annexin V‐FITC/PI Apoptosis Kit (Elabscience, E‐CK‐A211); Aspartate aminotransferase Assay Kit (Nanjing Jiancheng Bioengineering Institute, C010‐2‐1); Alanine aminotransferase Assay Kit (Nanjing Jiancheng Bioengineering Institute, C009‐2‐1); Urea Assay Kit (Nanjing Jiancheng Bioengineering Institute, C013‐2‐1); Myeloperoxidase Activity Assay Kit (Elabscience, E‐BC‐K074‐M); Chemiluminescent HRP Substrate (Millipore, WBKLS0100); DAPI (solarbio, C0065); RIPA Tissue/cell lysate solution (solarbio, R0020); Cell Counting Kit‐8 (Dojindo, CK04); Immunohistochemistry staining kit (Bioss, SP‐0023); 0.25% Trypsin‐EDTA (Gibco, 25200‐072); Fetal Bovine Serum (Procell, 14210–500); DMEM High‐glucose medium (Solarbio, 12100); TNF‐α, Human (GenScript, Z01001‐10); LPS (Escherichia coli O55:B5) (Sigma, L2880); Suramin (GlpBio, GC16832); TNF‐α ELISA MAXTM Deluxe Set (BioLegend, 430904); IL‐6 ELISA MAXTM Deluxe Set (BioLegend, 431304); IL‐1β ELISA MAXTM Deluxe Set (BioLegend, 432604); FITC anti mouse F4/80 (Biolegend, 123107); APC anti mouse CD86 (Biolegend, 105113).

### Animals

All animal studies were conducted in full compliance with the protocol approved by the Nankai University Experimental Animal Ethics Committee (NKUIRB2020050). We used C57BL/6J male mice, 6–8 weeks old, weighing 20–25 g. All mice were housed in the Experimental Animal Center, at Nankai University. All animals were housed with food and water available ad libitum in a 12 h light‐dark environment at 20–22 °C and 30–70% humidity.

### Cell Survival Assay

The cell survival assay was performed using the Cell Counting Kit‐8 according to the manufacturer's instructions. Luminescence was recorded with a TECAN plate reader.

### Animal Model of LPS‐induced Sepsis and Survival

For LPS‐induced sepsis model experiments, 25 male C57BL/6J mice, 6–8 weeks old, weighing 20–25 g, were randomly divided into five groups (*n =* 5 in each group), and survival was examined. The abdominal cavity was injected with 40 mg kg^−1^ LPS to induce sepsis. 30 min later, mice in group 1 were injected subcutaneously with 0.9% NaCl, mice in groups 2–4 were injected subcutaneously with **CV122** (20 mg kg^−1^), **CV122** (30 mg kg^−1^), and **CV122** (40 mg kg^−1^), respectively, and mice in group 5 were injected subcutaneously with Suramin (40 mg kg^−1^). The status of the mice was observed and recorded every day.

### Animal Model of CLP‐Induced Sepsis and Survival1

For CLP‐induced sepsis model experiments,^[^ ^44^ ^]^ fifteen male C57BL/6J mice, 6–8 weeks old, weighing 20–25 g, were randomly divided into three groups, and their initial body weight was measured and recorded. After the mice were anesthetized, their abdominal hair was shaved, the mouse's abdominal skin was disinfected with 75% alcohol, and ≈1–2 cm of the skin was cut with scalpel scissors. Ligated with a 4‐0 suture at a position 1/2 of the cecum length. The cecal contents were gently pushed to the distal end with tweezers, and the cecum was punctured with a 21G needle and then back and forth twice. Subsequently, a small amount of fecal drop was squeezed from the pinhole. The cecum was then placed back into the abdomen and the incision was closed with a 6‐0 suture. 30 min later, mice in the administration group were subcutaneously injected with **CV122** (40 mg kg^−1^).

### Animal Model of CCHFV‐Infected and Survival

CCHFV and IFNAR^−/−^ mice on the C57BL/6 (7‐8 weeks, male) background were from the State Key Laboratory of Virology, Wuhan Institute of Virology, Chinese Academy of Sciences, and the whole experiment was performed in the P3 laboratory. The experimental mice were divided into two groups: 1) Control (0.9% NaCl): 5 males (IFNAR^−/−^) and 2) Treatment group (**CV122**): 5 males (IFNAR^−/−^). Virus‐infected mice: CCHFV, 5000TCID_50_, **CV122** (20 mg kg^−1^) was administered by subcutaneous treatment at 2, 3, 4, and 5 days after infected CCHFV respectively, Control mice were injected with equal doses of 0.9% NaCl, 100 µL for each mouse. Mice were monitored daily for their body weights and survival rates.

### Cytokine Antibody Array

For LPS‐induced sepsis model experiments, twenty male C57BL/6J mice were randomly divided into four groups (*n* = 5 in each group). Mice in group 1 were injected with 0.9% NaCl, while mice in groups 2–4 were injected intraperitoneally with LPS (20 mg kg^−1^). After 30 min, mice in group 3 were injected with 40 mg kg^−1^ **CV122** and mice in group 4 were injected with 40 mg kg^−1^ Suramin. After 3 h, blood was taken and the lungs, liver, spleen, kidney heart, and brain were taken. For CLP experiments, the mice were euthanized 24 h after the establishment of the sepsis model. Blood samples were collected from the eyeballs of mice, the lungs were taken. These organs were ground at 0.1 g mL^−1^ in PBS, centrifuged and the supernatant collected. Serum and organ tissue supernatants were used to detect cytokine levels using the mouse TNF‐α, IL‐6, IL‐1β ELISA MAXTM Deluxe Set.

### Micro‐CT

For LPS‐induced sepsis model experiments, mice were anesthetized at 12 h of LPS injection. Micro‐CT images of the lungs of mice were acquired using the SkyScan 1276 high‐resolution µCT system at 50 kVp, 180 µA, and 150 mGy. After radiographic data of CT were acquired, images of the lungs of mice were reconstructed by 2D image analysis software provided by CTvox, and 3D images were reconstructed using 3D Slicer.

### Tissue Injury

For LPS‐induced and CLP‐induced sepsis model experiments, the mice were euthanized 24 h after the establishment of the sepsis model. Blood samples were collected from the eyeballs of these mice. The lungs, kidneys, livers, and spleen were harvested from mice and fixed in 10% formalin for 24 h before paraffin fixation. Tissues were sectioned (4 µm) and stained with hematoxylin and eosin (H&E). For CCHFV‐infected mice model experiments, the mice were euthanized and collected spleen 7 d after infection with CCHFV. Blood serum was collected to measure liver injury markers (AST, ALT), kidney injury markers (BUN), and lung injury markers (MPO) activity in lung tissues using kits purchased from Nanjing Jiancheng Bioengineering Institute according to the manufacturer's instructions.

### Lung Wet–Dry Weight Ratio

Fresh lung from each group of mice was removed, drained, and weighed to obtain a “wet” weight, and then dried in a 60 °C oven for 48 h to obtain a “dry” weight. The ratio of two lung tissue weights, the wet–dry ratio (W–D), was calculated to reflect the degree of pulmonary edema of mice.

### Flow Cytometry

Each group of mice was euthanized 24 h after LPS injection, spleens were harvested, red blood cells were lysed to detect splenocyte apoptosis, and the ratio of CD3^+^CD4^+^ T cells, CD3^+^CD8^+^ T cells, CD3^−^CD19^+^ B cells in spleen cells and lymph nodes quantified. Antibodies were: FITC‐Annexin V and PI; APC‐CD3, FITC‐CD4, PE‐CD8a, and PE‐CD19. Each group of mice was euthanized 12 h after LPS injection, Lung, kidney, and spleens were harvested, red blood cells were lysed to detect the ratio of macrophages in multiple organs, Antibodies were: FITC anti‐mouse F4/80; APC anti‐mouse CD86. Flow cytometry was used to quantify the adhesion molecules ICAM‐1 and VCAM‐1 on the surface of RAW264.7 cells and HUVECs. **CV122** (50 µM) was incubated with the cells for 3 h, LPS (5 µg mL^−1^) and TNF‐α (20 ng mL^−1^) were used to stimulate the cells for 1 h, and the cells were collected. FITC anti‐mouse CD54 antibody and AF647 anti‐mouse CD106 antibody were incubated for 30 min at room temperature in the dark, PBS was added at 1000 rpm, and the cells were centrifuged for 7 min. A 200‐mesh copper mesh was used to filter the cells into a flow cytometer, and a flow cytometer (BD Biosciences) was used for detection.

### Homogeneous Time‐Resolved Fluorescence Technology

Active HPA was diluted with Buffer A to 120 ng mL^−1^, biot‐HS‐K was diluted with Buffer B (pH 5.5) to 1.4 ng mL^−1^, and SA‐d2 was diluted with Buffer C (pH 7.5) to 1 µg mL^−1^, **CV122** samples were diluted with Buffer A to different concentration gradients. Add 4 µL of sample solution and 3 µL of HPA solution or Buffer A to the micro‐96‐well plate. After incubating at 37 °C for 10 min, the enzyme reaction was started by adding 3 µL of biot‐HS‐K and incubating at 37 °C for 30 min. Then add 10 µL of SA‐d2 solution or Buffer C. After incubating for 15 min at room temperature, the HTRF signal was detected by a microplate reader, which was excited at 384 nm and emitted at 620 nm and 665 nm. The fluorescence intensity ratio of 665 nm/620 nm was used to calculate the average energy transfer rate (Δ*F*%) of each sample. The positive control system composed of biot‐HS‐K and SA‐d2 should produce the largest Δ*F*%, while the background fluorescence intensity of the negative control (biot‐HS‐K) should produce the smallest Δ*F*%.^[^ ^45^ ^]^

### Immunofluorescence

Lungs were harvested from LPS‐induced and CLP‐induced sepsis model mice, and frozen sections were prepared. HUVECs and RAW264.7 macrophages were incubated with **CV122** for 3 h, and TNF‐α (20 ng mL^−1^) and LPS (5 µg mL^−1^) were used to stimulate cells for 1 h. Lung tissue, HUVECs, and RAW264.7 macrophages were fixed with 4% paraformaldehyde for 10 min before permeabilization with 0.5% Triton X‐100 for 10 min. The membrane was sealed with 4% skimmed milk powder and incubated at room temperature for 50 min. Primary antibodies were rabbit anti‐heparanase polyclonal antibodies overnight at 4 °C. The diluent of the secondary antibody FITC‐WGA‐lectin was kept at room temperature and protected from light for 1 h. Nuclei were stained with DAPI. After 120 s, images were acquired with a confocal microscope (LSM 800, Zeiss, Germany).

### Immunohistochemistry

The lung, kidney, and liver tissues of mice were fixed in paraformaldehyde. Sections underwent endogenous peroxidase quenching and antigen retrieval followed by incubation with primary antibodies or isotype‐matched controls. Slides were incubated in horseradish peroxidase (HRP)‐conjugated antibodies followed by visualization with HRP‐AB‐C substrate.

### Western Blotting

**CV122** (50 µM) was incubated with HUVECs and RAW264.7 cells for 3 h, and LPS (5 µg mL^−1^) and TNF‐α (20 ng mL^−1^) were incubated for 1 h. Total protein was extracted with RIPA tissue/cell lysate solution. Lung tissues were collected and homogenized in cold RIPA buffer to obtain proteins. The BCA method was used to quantify protein. After full denaturing, proteins were separated by 10% SDS‐PAGE and transferred to PVDF membrane. Membranes were blocked in 5% skimmed milk powder in PBS for 60 min. Primary antibodies were rabbit anti‐heparanase polyclonal antibody diluted 1:1000, while secondary antibodies were HRP‐conjugated Affinipure goat anti‐rabbit IgG. Anti‐GAPDH antibodies diluted 1:10000 were used as a reference with secondary HRP‐conjugated Affinipure goat anti‐mouse IgG antibodies. Blots were developed with a chemiluminescent HRP substrate, and the protein bands were detected with a chemiluminescence imager (MiniChemi610).

### Atomic Force Microscopy

HUVECs and RAW264.7 cells in the logarithmic growth phase were digested, centrifuged, and then plated in a six‐well plate at a density of 1 × 10^5^ cells per well. **CV122** was incubated for 3 h, and cells were stimulated with LPS (5 µg mL^−1^) and TNF‐α (20 ng mL^−1^) for 5 h. Cell‐covered slides were carefully removed and placed on the stage of the atomic force microscope with needle parameters: elastic coefficient 0.4 N M^−1^, sensitivity 65.63 nm V^−1^, with otherwise the usual atomic force microscope (Bruker, Billerica, MA) parameters used.

### Electron Microscopy

The endothelial glycocalyx was detected by electron microscopy. Mice were dissected and a needle was inserted into the left ventricle of the heart and perfused with lanthanum aldehyde, a solution consisting of 2% glutaraldehyde, 2% sucrose, 0.1 M sodium phthalate buffer (pH 7.3), and 2% lanthanum nitrate. Lanthanum was a trivalent cation that binds polysaccharide‐protein complexes with negatively charged glycoprotein parts.^[^ ^46^ ^]^ The organs were cut into 1 mm^3^ pieces and then the samples were fixed in lanthanide aldehyde fixative. Fixed samples were prepared with 2.5% glutaraldehyde and phosphate buffer for 4 h or longer, rinsed three times with 0.1 M phosphorinse; fixed with 1% osmium acid fixative for 1–1.5 h, and rinsed with 0.1 m phosphorinse three times. After dehydration and curing, sections were made in a 70 nm copper mesh condition using a Reichert‐Jung E ultrathin slicer (Austria). Sections were stained with urium acetate‐lead citrate double dye and finally, images were with a JEM1200 electron microscope.

### Statistical Analyses

Data were analyzed using ZENblue, ImageJ, NanoScope Analysis 1.8, iViewer, and GraphPad Prism 8. GraphPad Prism 8 was used for *t*‐tests, and the mean ± SD was used to present continuous data. Each experiment was repeated at least three times. *p < 0.05, **p < 0.01, ***p < 0.001, ****p < 0.0001, ns, not significant.

## Conflict of Interest

The authors declare no conflict of interest.

## Author Contributions

D.W. and K.W. contributed equally to this work. D.Y.W. and H.Z.J. performed most of the experiments, analyzed data, and wrote the manuscript. K.X.W., Q.T.L., F.Y., M.Y.L., G.Q.Z., K.F., K.W., X. D., and M.L.W. contributed to experiments. W.Z., F.Y., G.Q.Z., Y.H.L., H.M.Z., S.Y., T.H.L., P.G., and P.G.W. provided advice on the manuscript. H.Z.J., F.Y., and W.Z. designed experiments, supervised the study, reviewed, and edited the manuscript.

## Supporting information

Supporting Information

## Data Availability

The data that support the findings of this study are available from the corresponding author upon reasonable request.
