# Supplementary material for: A Novel Drug Candidate for Sepsis Targeting Heparanase by Inhibiting Cytokine Storm
Source: Adv Sci (Weinh). 2024 May 29;11(29):2403337. doi: 10.1002/advs.202403337 (PMC11304236; doi:10.1002/advs.202403337)
Supplement: Supplementary file 1 — Supporting Information [file ADVS-11-2403337-s001.pdf]

ADVANCED SCIENCE
Open Access

Supporting Information

for *Adv. Sci.*, DOI 10.1002/advs.202403337

A Novel Drug Candidate for Sepsis Targeting Heparanase by Inhibiting Cytokine Storm

Danyang Wang, Kaixuan Wang, Qiutong Liu, Mingyang Liu, Guoqiang Zhang, Ke Feng, Kun Wang, Xianwei Ding, Haomiao Zhu, Song Yang, Yonghui Liu, Tiehai Li, Peng Gong, Manli Wang, Peng George Wang, Hongzhen Jin, Wei Zhao* and Fan Yu**

Supplementary information

A Novel Drug Candidate for Sepsis Targeting Heparanase by Inhibiting Cytokine Storm

Danyang Wang†, Kaixuan Wang†, Qiutong Liu, Mingyang Liu, Guoqiang Zhang, Ke Feng, Kun Wang, Xianwei Ding, Haomiao Zhu, Song Yang, Yonghui Liu, Tiehai Li, Peng Gong, Manli Wang, Peng George Wang, Hongzhen Jin, Wei Zhao* and Fan Yu**

A Novel Drug Candidate for Sepsis Targeting Heparanase by Inhibiting Cytokine Storm

Danyang Wang[1]†, Kaixuan Wang[1]†, Qiutong Liu[1], Mingyang Liu[1], Guoqiang Zhang[1], Ke Feng[1], Kun Wang[1], Xianwei Ding[1], Haomiao Zhu[1], Song Yang[2], Yonghui Liu[1], Tiehai Li[3], Peng Gong[4], Manli Wang[4], Peng George Wang[5], Hongzhen Jin[2]*,Wei Zhao[1]* and Fan Yu[2]*

1. State Key Laboratory of Medicinal Chemical Biology, College of Pharmacy, College of Life Sciences, Key Laboratory of Molecular Drug Research and KLMDASR of Tianjin, Nankai University, Tongyan Road, Haihe Education Park, Tianjin 300350, China
2. School of Health and Life Sciences, University of Health and Rehabilitation Sciences, Qingdao 266113, China
3. Carbohydrate-Based Drug Research Center, Shanghai Institute of Materia Medica, Chinese Academy of Sciences, Shanghai 201203, China
4. State Key Laboratory of Virology, Wuhan Institute of Virology, Chinese Academy of Sciences, Wuhan 430071, China.
5. School of Medicine, Southern University of Science and Technology, Shenzhen 518000, China

*Corresponding author. Email:
jinhongzhen@uor.edu.cn
wzhao@nankai.edu.cn
yufan@uor.edu.cn
†These authors contribute equally.

Supplementary Materials

Figure S1. Synthesis of building blocks.

Figure S1-1. Synthesis of **CV122**.

Figure S2. Characterization of **CV122**.

Figure S3. Inflammatory factors in LPS-induced septic mice.

Figure S4. The mechanism of action of **CV122** on Raw264.7.

Figure S5. CV122 inhibits HPA levels and reverses immunosuppression in LPS-induced severe septic mice.

Figure S6. M1 macrophages in multiple organs of LPS-induced septic mice.

A

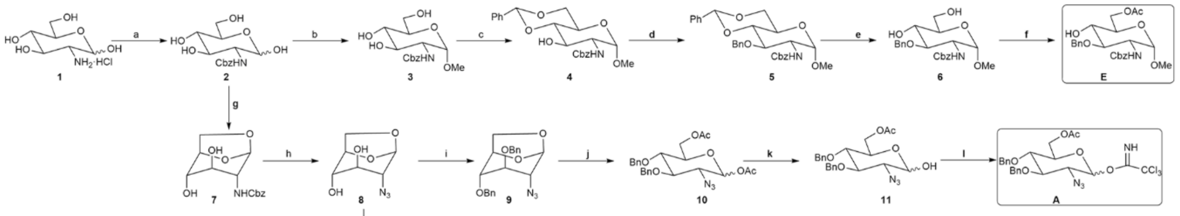

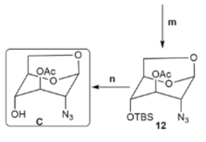

a) CbzCl, NaOH, H_2O, r.t., 60%; b) MeOH, AcCl, 60 °C, 70%; c) $PhCH(OMe)_2$, CSA, DMF, r.t., 76%; d) BnBr, NaH, THF, 0 °C to r.t., 70%; e) 80% AcOH, 90 °C, 87%; f) Ac_2O, Et_3N, DCM, 0 °C, 95%; g) 1) TsCl, Py, 4A MS, -20 °C to -10 °C, 3-5 h; 2) EtOH, DBU, NaI, 44% for two steps; h) 1) 4 atm H_2, Pd/C, MeOH; 2) TfN_3, $CuSO_4$, Et_3N, MeOH, 85% for two steps; i) BnBr, NaH, DMF, 89%, 0 °C; j) Ac_2O, TBSOTf, 0 °C, 100%; k) $BnNH_2$, THF, r.t., overnight, 85%; l) Cl_3CCN, K_2CO_3, DCM, r.t., 100%; m) 1) TBSCl, Imidazole, DMF; 2) Ac_2O, Py, 0 °C, 65% for two steps; n) TBAF, THF, r.t., overnight, 95%.

B

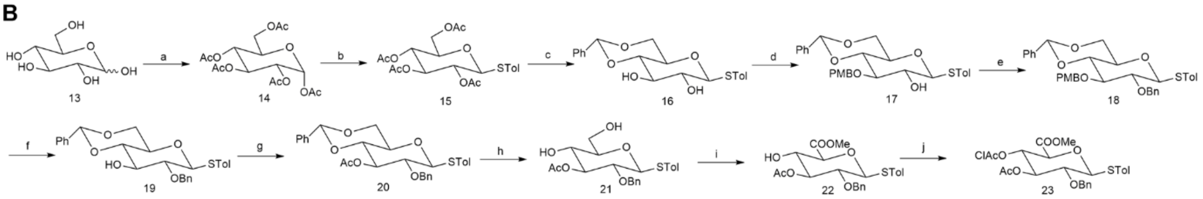

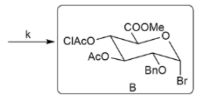

a) Ac_2O, $HClO_4$, 77%; b) TolSH, BF3¡¤Et2O, DCM, 86%; c) 1) MeONa, MeOH, DCM; 2) Benzaldehyde Dimethylacetal, CSA, DMF, 83% for two steps; d) 1) Bu_2SnO, MeOH, reflux; 2) PMBCl, CSF, DMF, 90 °C, 75% for two steps; e) BnBr, NaH, DMF, 92%; f) DDQ, DCM, H_2O, 78%; g) Ac_2O, Et_3N, DCM, 98%; h) 80% AcOH, 90 °C, 76%; i) 1) 2,2,6,6-Tetramethylpiperidinooxy(TEMPO), Iodobenzene diacetate, DCM, H_2O; 2) MeI, $KHCO_3$, DMF, 57% for two steps; j) $ClAc_2O$, Py, DCM, 76%; k) IBr, DCM, 76%.

C

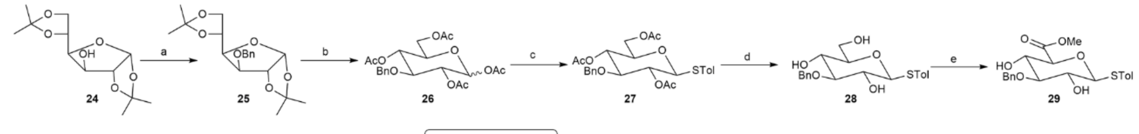

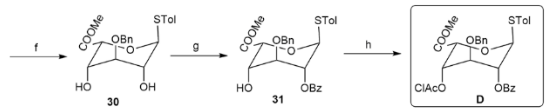

a) BnBr, NaH, DMF 0 °C; b) 1) H^+ resin, H_2O, 70 °C; 2) Ac_2O, Py, r.t., 46% for three steps; c) TolSH, $BF_3 \cdot Et_2O$, DCM, r.t., 85%; d) MeONa, MeOH, r.t., 100%; e) 1) 2,2,6,6-Tetramethylpiperidinooxy(TEMPO), Iodobenzene diacetate, DCM, H_2O r.t.; 2) MeI, $KHCO_3$, DMF, r.t., 51% for two steps; f) MeONa, MeOH, r.t., 70%; g) 1) Bu_2SnO, MeOH, 70 °C; 2) BzCl, Et_3N, THF, 0 °C, 69% for two steps; h) $ClAc_2O$, Py, DCM, 0 °C, 89%.

D

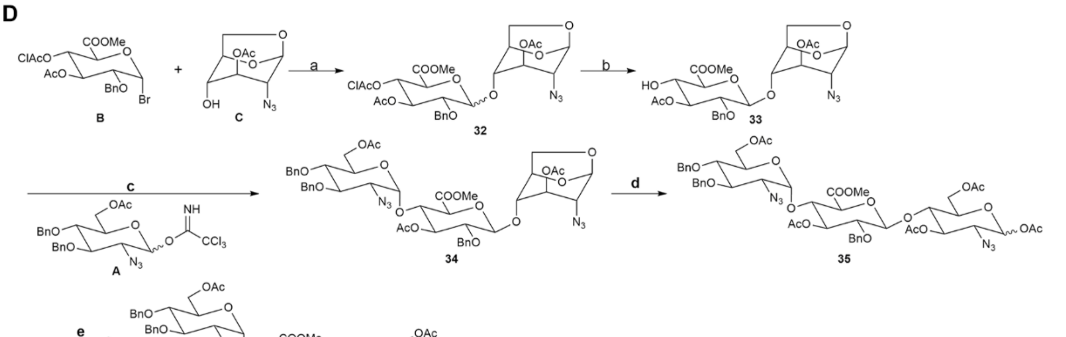

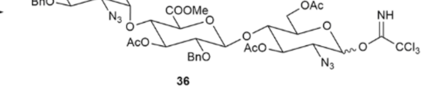

a) Ag_2CO_3, AgOTf, 4 A MS, DCM, r.t., overnight; b) Thiourea, $CHCl_3$, MeOH, 60 °C, 42% for two steps; c) Toluene, TfOH, 4 A MS, -20 °C to r.t., 87%; d) Ac_2O, TBSOTf, 0 °C, 100%; e) 1) $BnNH_2$, THF, r.t., overnight; 2) Cl_3CCN, K_2CO_3, DCM, r.t., 72% for two steps.

E

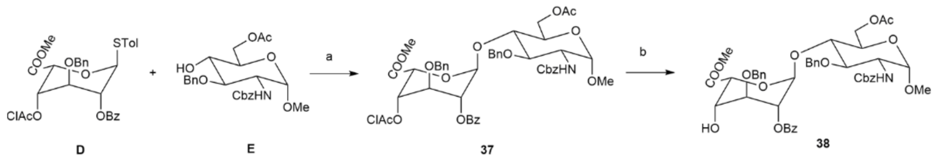

a) Ph_2SO, Tf_2O, 4 A MS, DCM, 93%; b) Thiourea, $CHCl_3$, MeOH, 60 °C, 86%.

F

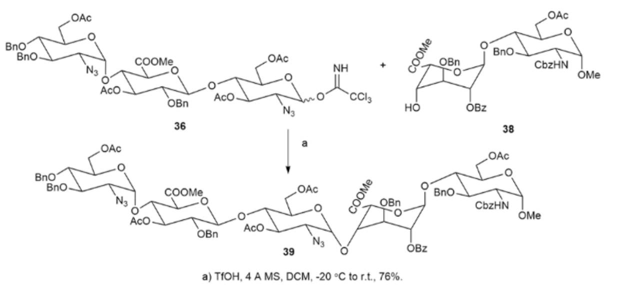

a) TfOH, 4 A MS, DCM, -20 °C to r.t., 76%.

Figure S1. Synthesis of building blocks. A) Synthesis of glucosamine A, C and E. B) Synthesis of GlcA donor B. C) Synthesis of IdoA donor D. D) Synthesis of trisaccharide donor 36. E) Synthesis of disaccharide acceptor 38. F) Synthesis of pentasaccharide 39.

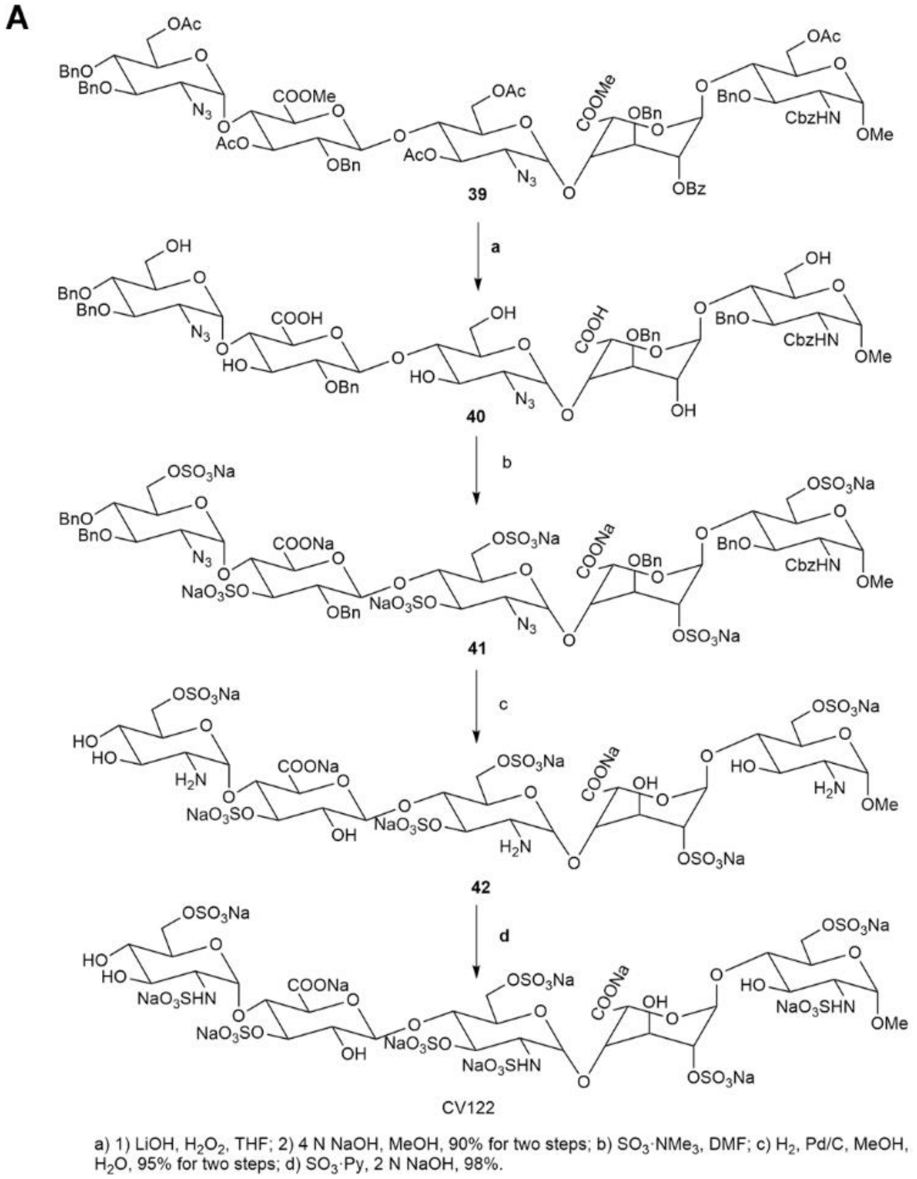

Figure S1-1. Synthesis of **CV122**.

^{1}H NMR spectrum of compound **CV122** in D_2O (400 MHz).

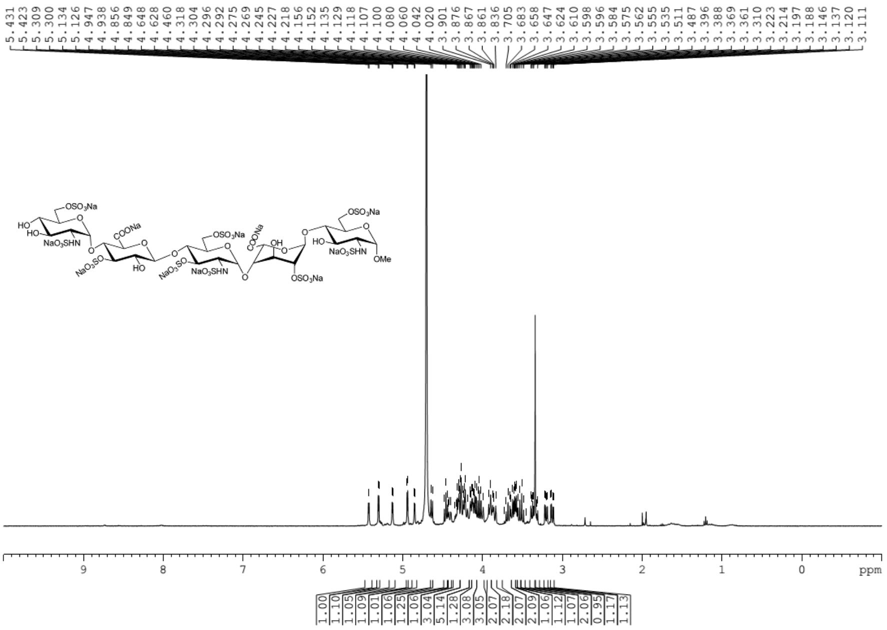

^{13}C NMR spectrum of compound **CV122** in D_2O (100 MHz).

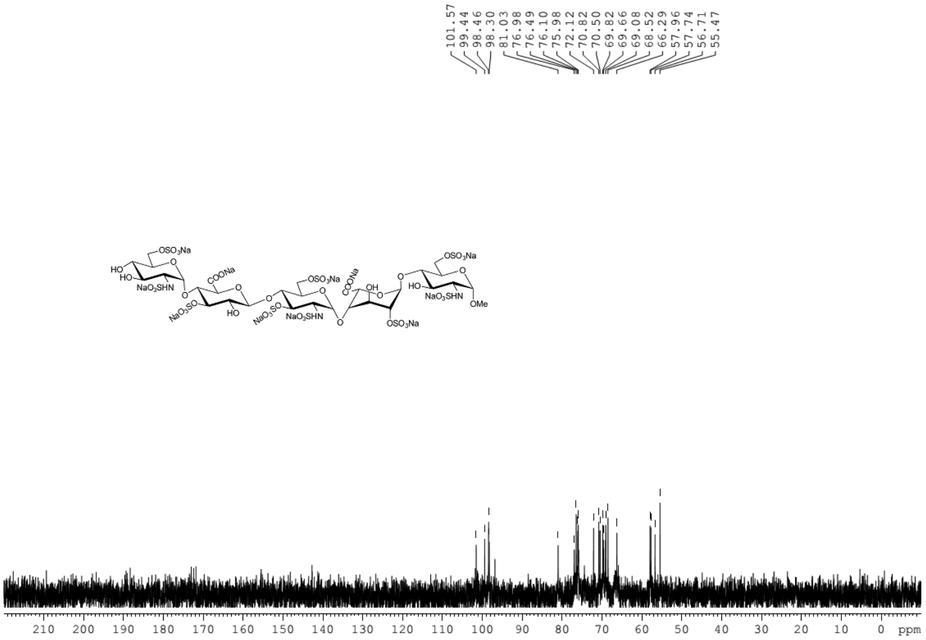

Figure S2. Characterization of **CV122**. ^{1}H NMR (400 MHz, D_2O) δ 5.45 (d, *J* = 3.3 Hz, 1H), 5.33 (d, *J* = 3.3 Hz, 1H), 5.21 (d, *J* = 2.9 Hz, 1H), 5.08 (d, *J* = 2.9 Hz, 1H), 4.99 (d, *J* = 3.4 Hz, 1H), 4.72 (s, 3H), 4.53 (t, *J* = 7.8 Hz, 1H), 4.44 (s, 1H), 4.41 – 4.08 (m, 11H), 3.92 (dd, *J* = 19.1, 9.2 Hz, 4H), 3.79 – 3.60 (m, 4H), 3.57 (d, *J* = 9.6 Hz, 1H), 3.47 – 3.31 (m, 4H), 3.30 – 3.22 (m, 1H), 3.22 – 3.11 (m, 1H). ^{13}C NMR (100 MHz, D_2O) δ 174.87, 174.29,101.74, 99.38, 98.57, 98.30, 97.31, 80.65, 76.97, 76.45, 76.17, 76.13, 75.78, 75.35, 74.69, 72.12,

70.73, 70.59, 69.82, 69.24, 69.11, 68.53, 68.50, 66.64, 66.27, 65.88, 57.92, 57.79, 56.74, 55.50. HRMS $[M–3Na]^{3-}$ m/z 586.5803(calcd for $C_{31}H_{45}N_3Na_8O_{52}S_9^{3-}$, 586.5801).

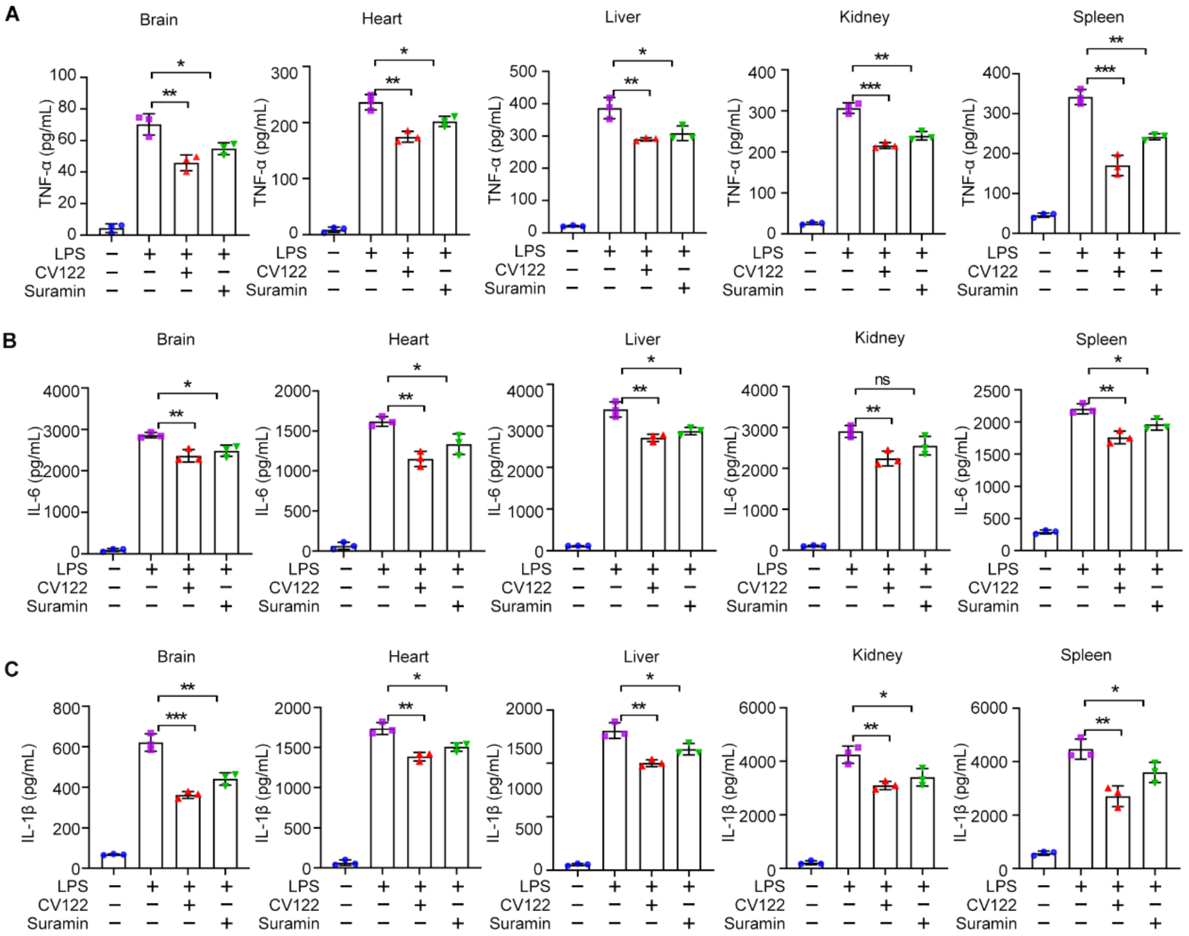

Figure S3. Inflammatory factors in LPS-induced septic mice. A) TNF-α in Brain, heart, Liver, Kidney and Spleen of mice. B) IL-6 in Brain, heart, Liver, Kidney and Spleen of mice. C) IL-1β in Brain, heart, Liver, Kidney and Spleen of mice. *p < 0.05, **p < 0.01, ***p < 0.001, ****p < 0.0001; ns, not significant. Error bars indicate SD.

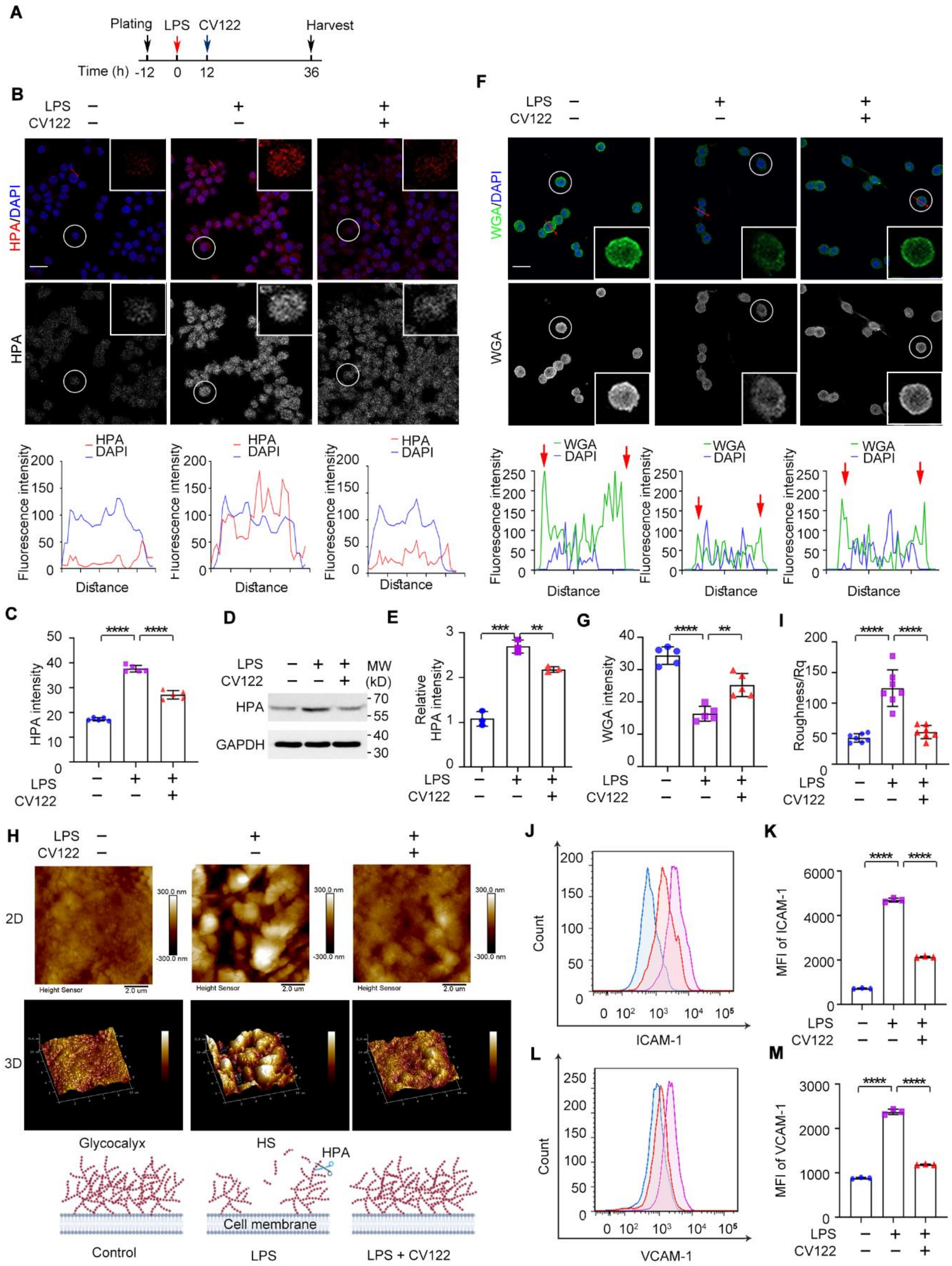

Figure S4. The mechanism of action of **CV122** on Raw264.7. A) Schematic diagram of cell experiment flow. B, C) HPA level was detected by immunofluorescence after LPS stimulation of Raw. Scale bars, 20 μm. D, E) Western blotting was used to detect HPA protein level in Raw, with GAPDH used as a housekeeping control. F, G) Laser confocal microscopy was used to detect changes in WGA-FITC fluorescence intensity in each group and after **CV122** treatment

on Raw. Scale bars, 10 μm. H, I) Atomic force microscopy was used to examine the effect of **CV122** on cell surface glycocalyx structure on Raw. Two- and three-dimensional structures on the cell surface were observed. J, K, L, M) ICAM-1 and VCAM-1 was detected by flow cytometry after LPS-stimulated Raw. *p<0.05, **p<0.01, ***$p < 0.001$, ****$p < 0.0001$; ns, not significant. Error bars indicate SD.

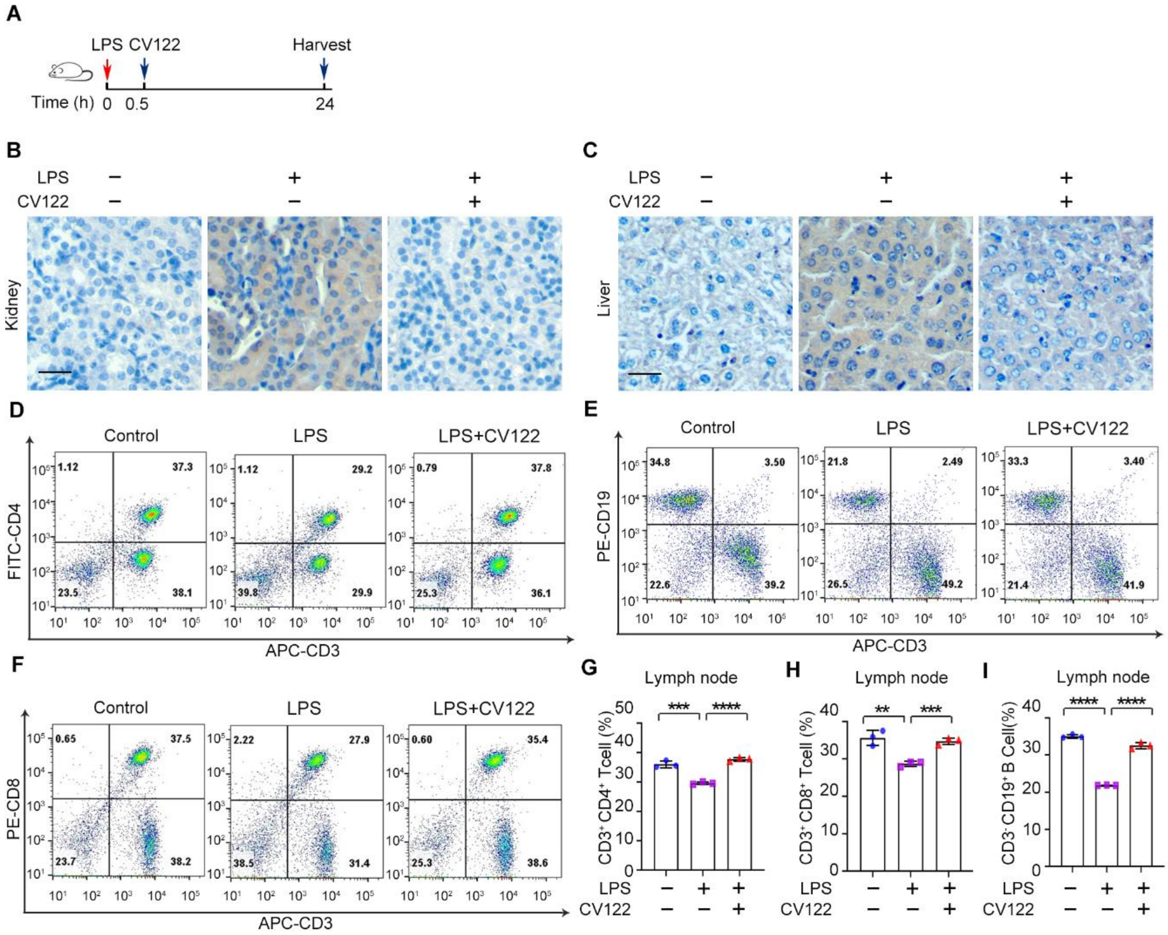

Figure S5. CV122 inhibits HPA protein levels in vivo and reverses immunosuppression in LPS-induced severe septic mice. A) Schematic diagram of mice experiment flow. B, C) Mouse Liver and Kidney tissue sections were stained with HPA antibody by Immunohistochemistry for detecting the level of HPA. D-I) Flow cytometric detection of $CD3^+CD4^+$ T cells, $CD3^+CD8^+$ T cells, $CD3^-CD19^+$ B cells in lymph node. Scale bars, 20 μm. **$p < 0.01$, ***$p < 0.001$, ****$p < 0.0001$; ns, not significant. Error bars indicate SD.

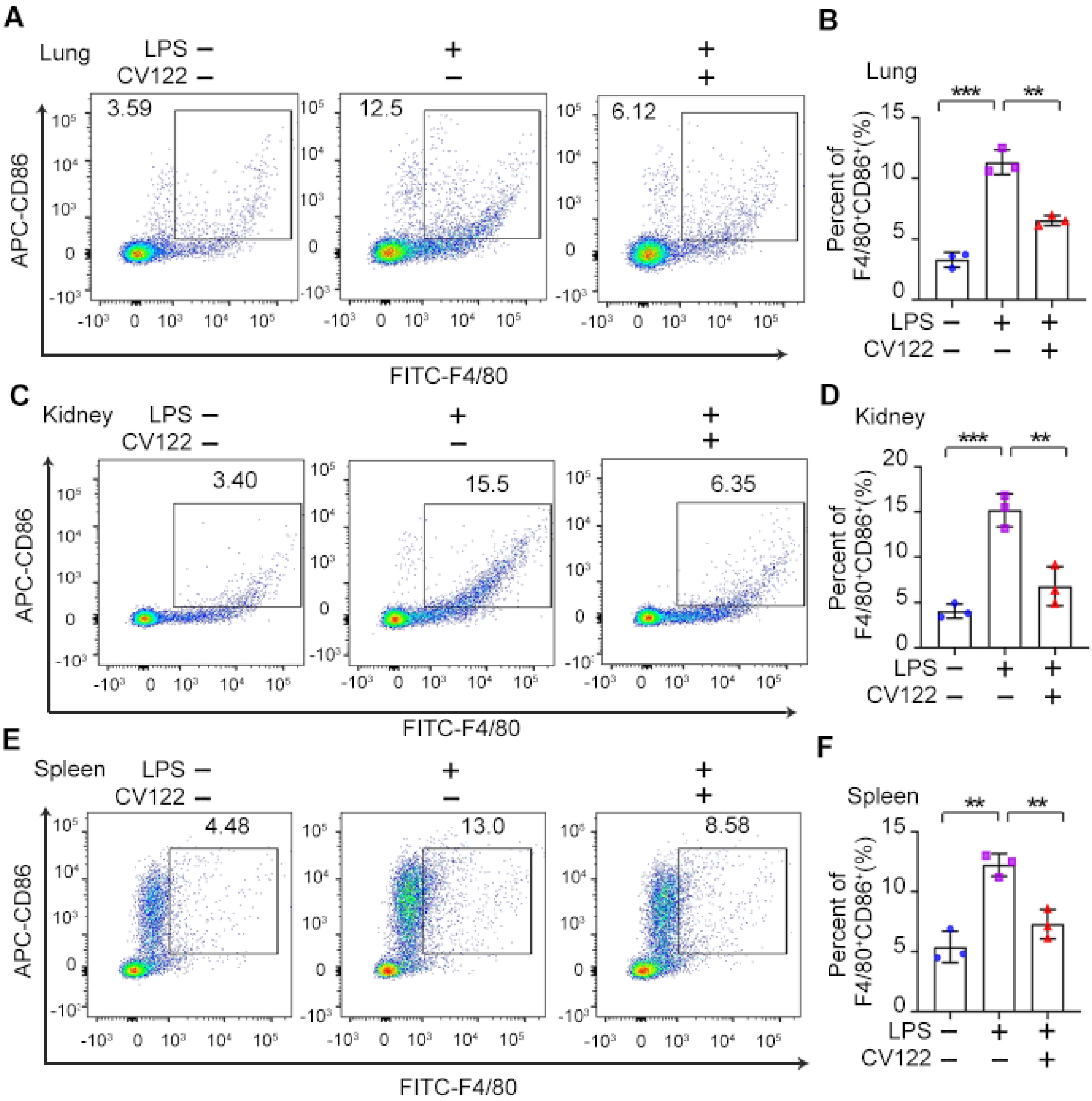

Figure S6. M1 macrophages in multiple organs of LPS-induced septic mice. A, B) M1 macrophages in Lung of LPS-induced septic mice by Flow cytometric detection of Percent of F4/80$^+$CD86$^+$. C, D) M1 macrophages in Kidney of LPS-induced septic mice by Flow cytometric detection of Percent of F4/80$^+$CD86$^+$. E, F) M1 macrophages in Spleen of LPS-induced septic mice by Flow cytometric detection of Percent of F4/80$^+$CD86$^+$. Mice were intraperitoneally injected with LPS (40 mg/kg). After 30 min, **CV122** was s.c. injected. Each group of mice were euthanized 12 h after LPS injection, Lung, kidney and spleens were harvested. All results are expressed as mean ± SD (n = 3), *p < 0.05,**p < 0.01, ***p < 0.001, ****p < 0.0001; ns, not significant. Error bars indicate SD.
